# Microtubule-associated protein 6 mediates neuronal connectivity through Semaphorin 3E-dependent signalling for axonal growth

**DOI:** 10.1038/ncomms8246

**Published:** 2015-06-03

**Authors:** Jean-Christophe Deloulme, Sylvie Gory-Fauré, Franck Mauconduit, Sophie Chauvet, Julie Jonckheere, Benoit Boulan, Erik Mire, Jing Xue, Marion Jany, Caroline Maucler, Agathe A. Deparis, Olivier Montigon, Alexia Daoust, Emmanuel L. Barbier, Christophe Bosc, Nicole Deglon, Jacques Brocard, Eric Denarier, Isabelle Le Brun, Karin Pernet-Gallay, Isabelle Vilgrain, Phillip J. Robinson, Hana Lahrech, Fanny Mann, Annie Andrieux

**Affiliations:** 1 INSERM, U836, F-38000 Grenoble, France; 2 Univ. Grenoble Alpes, Grenoble Institut Neurosciences, F-38000 Grenoble, France; 3 Aix-Marseille Université, CNRS, IBDM UMR 7288, 13288 Marseille, France; 4 Cell Signalling Unit, Children's Medical Research Institute, University of Sydney, Wentworthville, New South Wales 2145, Australia; 5 Centre Hospitalier Universitaire de Grenoble, IRMaGe, 38043 Grenoble, France; 6 CNRS, UMS 3552, 38042 Grenoble, France; 7 Lausanne University Hospital (CHUV), Department of Clinical Neurosciences (DNC), Laboratory of Cellular and Molecular Neurotherapies (LCMN), 1011 Lausanne, Switzerland; 8 Lausanne University Hospital (CHUV), Neuroscience Research Center (CRN), 1011 Lausanne, Switzerland; 9 INSERM, U1036, 38054 Grenoble, France; 10 CEA, iRTSV, F-38000 Grenoble, France; 11 CEA, LETI, CLINATEC, MINATEC Campus, F-38054 Grenoble, France

## Abstract

Structural microtubule associated proteins (MAPs) stabilize microtubules, a property that was thought to be essential for development, maintenance and function of neuronal circuits. However, deletion of the structural MAPs in mice does not lead to major neurodevelopment defects. Here we demonstrate a role for MAP6 in brain wiring that is independent of microtubule binding. We find that MAP6 deletion disrupts brain connectivity and is associated with a lack of post-commissural fornix fibres. MAP6 contributes to fornix development by regulating axonal elongation induced by Semaphorin 3E. We show that MAP6 acts downstream of receptor activation through a mechanism that requires a proline-rich domain distinct from its microtubule-stabilizing domains. We also show that MAP6 directly binds to SH3 domain proteins known to be involved in neurite extension and semaphorin function. We conclude that MAP6 is critical to interface guidance molecules with intracellular signalling effectors during the development of cerebral axon tracts.

## Abstract (web summary)

Loss of the structural microtubule-associated protein 6 (MAP6) leads to neuronal differentiation defects that are independent of MAP6's microtubule-binding properties. Here the authors establish a functional link between MAP6 and Semaphorin 3E signalling for proper formation of the fornix of the brain.

## Graphical abstract

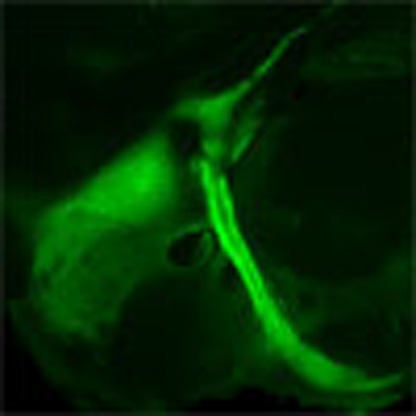

Appropriate adult brain function depends on the complex architecture of neuronal networks and synaptic connections that are elaborated during neurodevelopment. Convergent data show that abnormal anatomical and functional connectivity occurs in the brain of patients suffering from psychiatric diseases such as schizophrenia or bipolar disorder^1^,^2^,^3^,^4^,^5^,^6^. For proper brain connectivity, neuronal morphogenesis and differentiation requires a coordinated and dynamic organization of cytoskeletal elements, including the microtubular network. Microtubules (MTs) play key roles in these processes and determine axon formation^7^ and regulate the dynamics of dendritic spines^8^. Impairment of certain MT effectors causes severe neurodevelopmental disorders including lissencephaly, double cortex syndrome or microcephaly^9^,^10^,^11^. Among the MT regulators is a family of structural MAPs, including MAP1, MAP2, Tau and MAP6, which were first described as MT-stabilizing proteins^12^,^13^,^14^,^15^. Acute inhibition of any structural MAP in neuronal cells lines have led to impaired neuritic outgrowth providing the idea that MT stabilization would be crucial for normal neuronal differentiation during brain formation^16^,^17^,^18^,^19^. However and very strikingly, none of the knockout of structural *MAP* genes has led to lethal phenotypes due to massive neuronal differentiation impairments^20^,^21^,^22^,^23^. These findings raise the possibility of alternative roles of the structural MAPs during neurodevelopment, which still need to be identified.

Within the structural MAPs, MAP6 (also known as STOP for Stable-Tubule-Only-Polypeptide) was first characterized as a MT cold-stabilizing factor whose activity was inhibited by interaction with Ca^2+^-calmodulin^24^. The two main isoforms of MAP6 MAP6-E, which is expressed during neurodevelopment and in the adult brain, and MAP6-N, which is expressed postnatally^16^,^25^, exhibit pronounced axonal localization^26^,^27^. MAP6 null mice (MAP6 KO) are viable but display severe behavioural disorders associated with synaptic plasticity impairments and exhibit alterations in multiple neurotransmission^23^,^28^,^29^,^30^. Their behavioural and biological alterations resemble schizophrenia-related symptoms, and indeed, long-term treatment with antipsychotic drugs alleviates several of the behavioural and biological defects^23^,^31^,^32^. Their behavioural impairments may also rely on axonal disconnectivity, as some evidence points to structural changes in brain anatomy and connectivity of MAP6-KO mice^26^,^28^,^33^,^34^. However, a global and detailed analysis of brain alterations is as yet missing.

Here we examined the organization and structural integrity of the white matter in MAP6-KO mice. We found that MAP6 deletion causes severe hypoplasia of cerebral commissures and long-distance projecting axon tracts. The most striking defect is the absence of the post-commissural part of the fornix, producing neuronal disconnectivity between the hippocampus and the hypothalamus. Fornix formation is known to be dependent on signalling by the axon guidance cue Sema3E^35^,^36^. We demonstrated here that MAP6 plays a pivotal role downstream of Sema3E in promoting axonal growth and attractive guidance. MAP6-E binds to components of the Sema3E receptor complex and mediates interactions with downstream effectors through a proline-rich domain that is distinct from domains required of MT stabilization. Therefore, MAP6 is crucial for the establishment of functional Sema3E-dependent neural circuits via a MAP6 signalling function integrating semaphorin signalling, independent of microtubule binding. This reveals a previously unrecognized signalling function for MAP6, acting as a positional scaffold protein interfacing microtubule network and signalling events triggered by guidance cue receptors for the neuronal wiring.

## Results

### MAP6 deletion induces reduced brain volume

**Figure 1:**
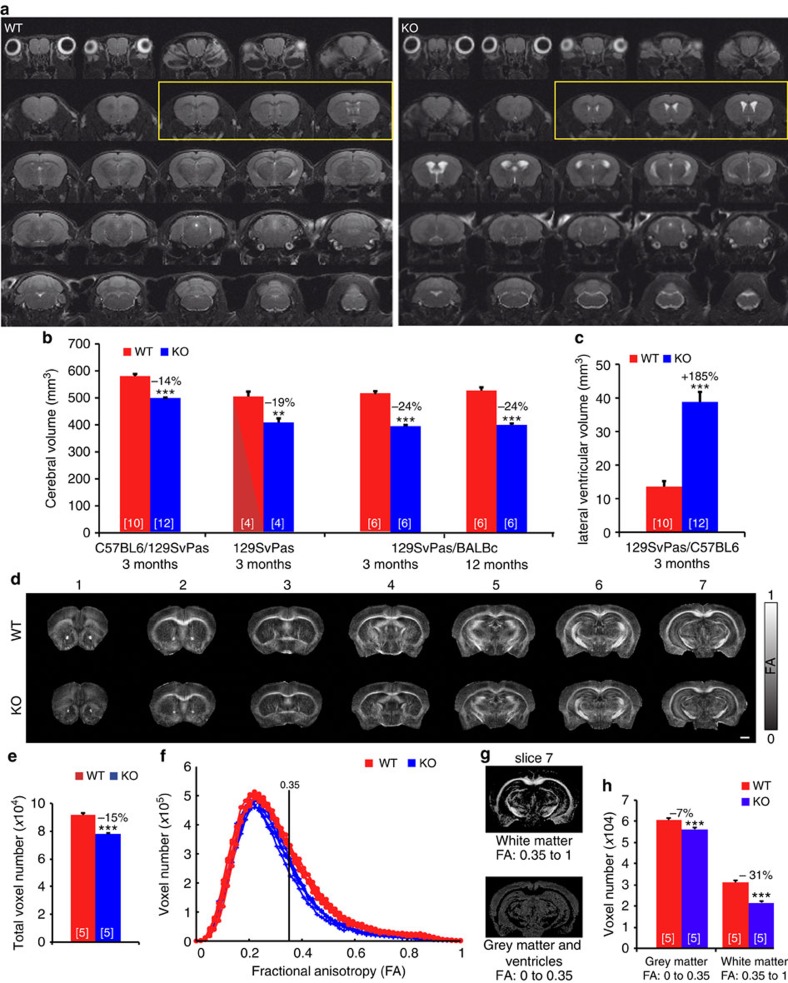
Reduced brain volume in MAP6-KO mice associated with deficits in axonal fibre tracts. (**a**) Representative galleries of T2-weighted MRI coronal sections used to determine the whole-brain and lateral ventricle volumes from WT and MAP6-KO mice. All sections were used to determine the cerebral volumes shown in **b**. Sections in the yellow frame were used to determine the lateral ventricle volume shown in **c**. (**b**) Cerebral volume in WT and MAP6-KO mice. A systematic decrease of MAP6-KO mice cerebral volume in different genetic backgrounds. (**c**) Histogram from MRI data showing ventricle enlargement in MAP6-KO mice. (**d**) Representative fractional anisotropy (FA) maps of the brains of WT and MAP6-KO. Scale bar, 1 mm. (**e**) Histogram showing a reduction of total voxel number of MAP6-KO brain. (**f**) Histograms showing the voxel number distribution according to FA values (the histogram of each mouse is individually plotted). FA threshold of 0.35 is used for white matter segmentation. (**g**) Typical FA maps segmentation from WT mouse. Image with FA>0.35 corresponds to the white matter imaging and FA<0.35 to grey matter and ventricles. (**h**) Histograms of voxel numbers corresponding to FA<0.35 and FA>0.35. In all panels, number of analysed mice is between brackets. Values are given as mean±s.e.m. Percentages of change between KO and WT are indicated.. **P<0.01; ***P<0.001 (Student's *t*-test).

To examine how MAP6 controls brain organization, we first used *in vivo* magnetic resonance imaging (MRI) to monitor the brain size of MAP6-KO mice from three genetic backgrounds (Fig. 1). Representative coronal T2-weighted images from wild-type (WT) and MAP6-KO mice were used to quantify the whole brain volume (Fig. 1a). Adult MAP6-KO mice from each genetic background showed reduced brain volume: 14% in homogeneous inbred C57BL6/129 SvPas-F1 mice, 19% in pure 129 SvPas mice and 24% reduction in heterogeneous non-inbred BALBc/129 SvPas-F2 mice, compared with WT littermates (Fig. 1b). A longitudinal study performed on BALBc/129 SvPas-F2 mice showed that the brain volume remained unchanged between 3 and 12 months of age, suggesting that the reduced brain volume was not progressive with age (Fig. 1b). Associated with the volume reduction was a global ventricle enlargement (Fig. 1a,c). All subsequent data were obtained using WT and KO mice on a homogeneous inbred 50% C57BL6/50% 129SvPas-F1 genetic background. We then used diffusion tensor imaging (DTI) to discriminate between grey matter (GM), mostly composed of neuronal cell bodies, and white matter (WM), mostly composed of myelinated axonal tracts. We focused on the forebrain that encompasses the major limbic cerebral structures implicated in the control of behaviours. Typical maps of fractional anisotropy (FA) values are shown in Fig. 1d, bright structures correspond to high FA values, that is, fibre bundles of WM and dark structures correspond to low FA values, that is, GM and ventricles. In all forebrain images of KO and WT mice, the brain was delineated and the total number of voxels (pixels x thickness of the slice) was calculated for both genotypes. We observed a 15% reduction of voxel number in the forebrain of MAP6-KO mice as compared with WT mice (Fig. 1e), which is in agreement with the 14% total brain volume reduction obtained using anatomical T2-weighed MRI (Fig. 1b). The reduced voxel number in MAP6-KO mice was not homogeneously distributed among FA values, being more pronounced for FA values ranging from 0.3 to 0.6 (Fig. 1f). WM can be discriminated from GM by selecting FA values above 0.35 (Fig. 1g) and in MAP6-KO brains the number of voxels are reduced by 31% (*P*=8.6 10^−3^) for FA values above 0.35 (WM) and by 7% (*P*=2.5 10^−5^) for FA values below 0.35 (GM) (Fig. 1h). Therefore, MAP6-KO mice exhibit reduction in whole brain volume that is associated with strong deficits in axonal fibre tracts.

### MAP6 is required for axonal tracts integrity

**Figure 2:**
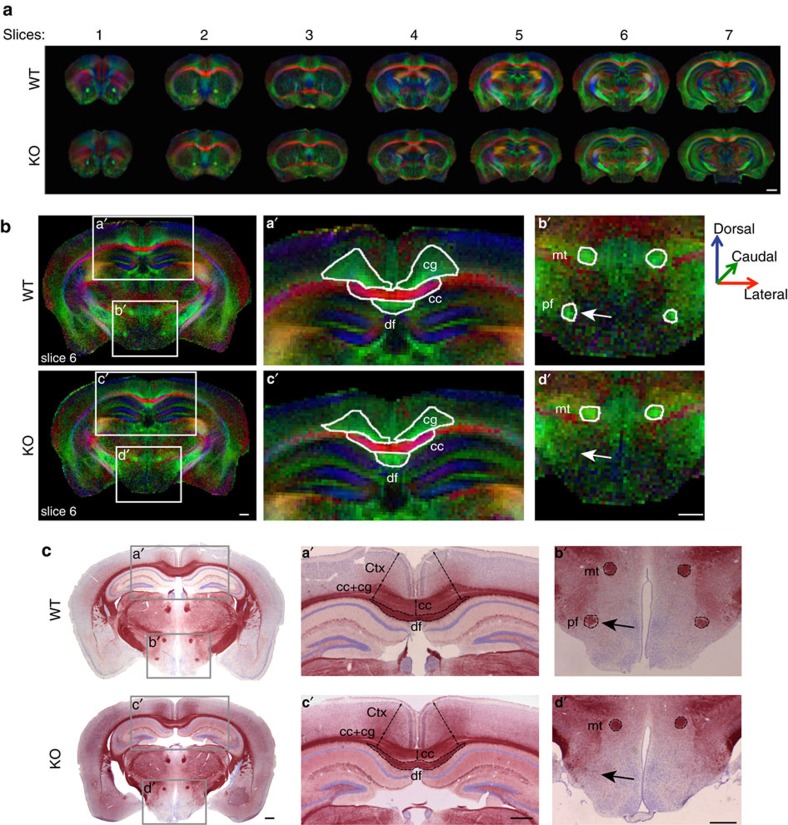
Axonal tracts are defective in MAP6-KO mice. (**a**) Representative galleries of FA colour maps in which the colour code indicates the spatial direction of the free diffusion along the axons with medial-lateral (red), ventral-dorsal (blue) and rostral-caudal (green) orientations. Scale bar, 1 mm. (**b**) Representative FA colour-maps (slices 6 from **a**) used to draw each axonal tract. FA values and region of interest (ROI) surface areas for each tract are reported in Table 1. Scale bar, 500 μm. (**c**) Representative coronal brain sections of WT and MAP6-KO forebrain stained with gold chloride and counterstained with cresyl violet used to measure the area and the thickness of axonal tracts and brain regions. Scale bar, 500 μm. Note in panels **b**′ and **d**′ that the post-commissural part of fornix (arrow, pf) is undetectable in the MAP6-KO brain slice after gold staining or DTI images. cc=corpus callosum; cg=cingulum; Ctx=cortex; df=dorsal fornix; mt=mammillary tract and pf=post-commissural fornix.

We next use the DTI colour map, which graphically illustrates the direction of fibres, to perform a detailed and global examination of axonal tracts within the forebrain (Fig. 2a,b, Table 1). Analysis of FA colour maps from MAP6-KO as compared with WT brains revealed a severe diminution of the size of the corpus callosum (Fig. 2b, Table 1, 25 to 35%), the cingulum (Fig. 2b, Table 1, up to 26%), the internal capsule (not shown, Table 1, 44%), the anterior commissure (not shown, Table 1, 26 to 27%,) and the cerebral peduncle (not shown, Table 1, 24 to 34%). Other tract sizes were less affected, such as the fimbria (not shown, Table 1, up to 11%) or unaffected, such as the mammillary tract or the dorsal fornix (Fig. 2b, Table 1). Thus, MAP6 deletion induced a non-uniform alteration of axonal tracts. To avoid a possible under-estimation of tract sizes due to reduction of FA values we performed a classical histological study on coronal forebrain brain sections to accurately measure fibre track size (Fig. 2c and data not shown). Slices were selected based on morphometric landmarks and stained for myelin with gold chloride and Nissl substance with cresyl violet (Fig. 2c). This analysis revealed that axonal tracts are differentially affected, with a severe reduction of the area for the corpus callosum (Fig. 2c, up to 32%, *P*=7 × 10^−5^), the anterior commissure (not shown, up to 61%, *P*=1.1 × 10^−05^) and of the internal capsule (not shown, up to 49%, *P*=3.5 × 10^−05^), whereas other tracks are less severely affected such as the fasciculus retroflexus (not shown, 29%, *P*=1.4 × 10^−3^) or the fimbria (not shown, up to 23%, *P*=4.0 × 10^−3^).

One striking observation of both FA colour maps and histological slices was the absence of the ventral part of the fornix, defined as the post-commissural fornix (pf) in MAP6-KO brains (Fig. 2b,c, arrows).

To assess fibre integrity, we compared the FA values between WT and MAP6-KO for each axonal tract (Table 1). FA values, for several axon tracts with reduced dimension, including the anterior commissure, the corpus callosum and the cingulum, are significantly affected in KO mice, with an average reduction of 15, 13 and 18%, respectively (Table 1). By contrast, FA values for the cerebral peduncle were not affected, despite size reduction. In addition, FA values of the dorsal fornix, the mammillary tract, the supraoptic and optic tracts were not affected. To properly interpret the FA changes, which are sensitive to myelin integrity, density and to the intra- and extracellular compartment volumes, we used transmission electron microscopy to investigate the microstructure of fibres from the corpus callosum. No ultrastructural axonal differences were evident (Supplementary Fig. 1a). Quantification of the thickness and perimeter of myelin sheaths as well as of myelinated axonal surfaces revealed no difference between WT and MAP6-KO axons (Supplementary Fig. 1b). Thus, FA changes indicate possible axonal density reduction with an increase of extra-axonal compartment volume or defasciculation defects in some tracts rather than myelination defects.

Altogether this analysis indicates that MAP6 deletion induces heterogeneous defects affecting both the size and the integrity of the axonal tracts and highlighted an apparently specific function of MAP6 in one specific tract: the post-commissural fornix.

### MAP6 is required for fornix integrity

**Figure 3:**
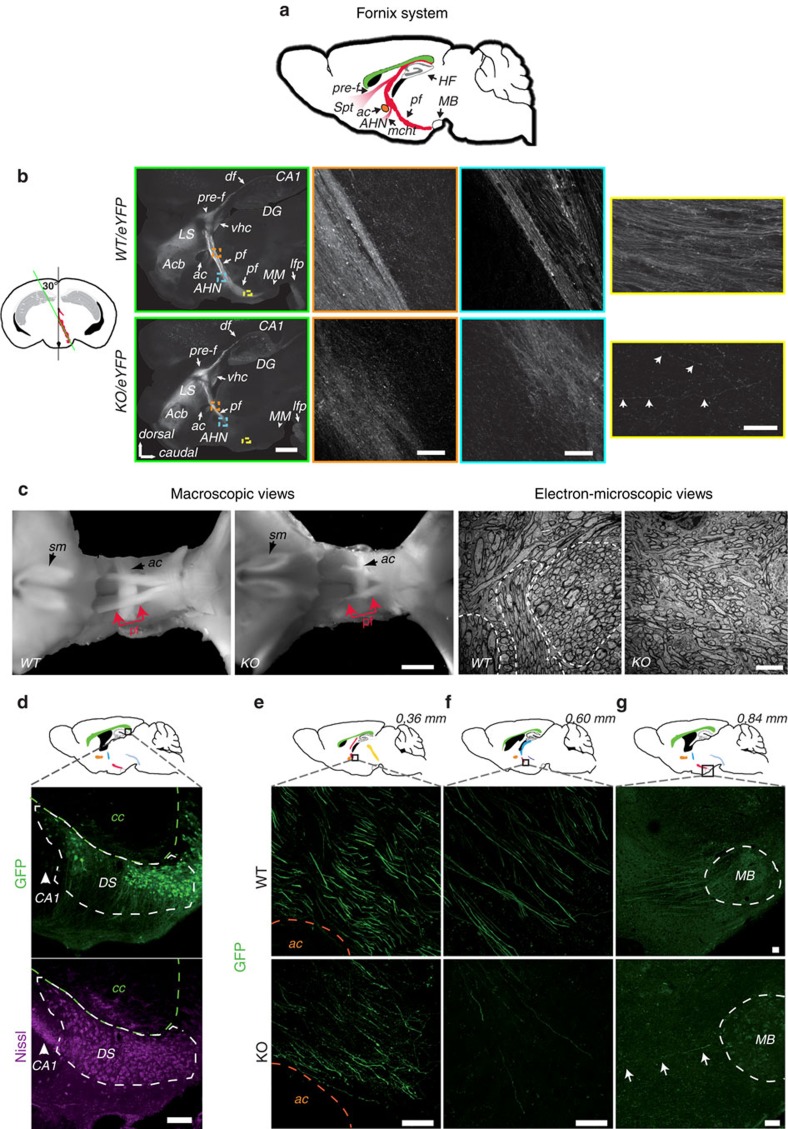
Disruption of the post-commissural fornix in MAP6-KO mice. (**a**) Schematic representation of the fornix system (in red) on a sagittal diagram. (**b**) Diagram showing in red, the projection stack of post-commissural fornix. The green line indicates the plane of the transverse sections (30°) shown in panels framed in green. Panoramic views of epifluorescence observed in WT/Thy1-eYFP-H (upper) and MAP6-KO/Thy1-eYFP-H brains (lower) are shown in green framed panels, scale bar, 1 mm. Orange, blue and yellow-framed panels represent confocal images corresponding to coloured squares drawn in the panoramic view. Images are the maximal projection stack of 10 confocal sections, separated by 0.95 μm. In yellow framed panels, arrows indicated the presence of some rare axonal projections reaching the mammillary body. Scale bars, 20 μm. (**c**) Dorsal macroscopic views showing the hypoplasia of MAP6-KO post-commissural fornices. Portions of post-commissural fornix used for electron microscopy studies are indicated in red, scale bar, 0.5 mm. Representative electron-microscopic views of transverse sections of post-commissure fornices of WT and MAP6-KO mice. In WT tight clusters of axons can be seen (dotted areas) not in MAP6-KO mice, scale bars, 5 μm. (**d**) A schematic sagittal section indicates the site of lentivirus stereotaxic injection in dorsal subiculum (square). Confocal images of the injection site (2 weeks post injection) show GFP-expressing pyramidal neurons in dorsal subiculum (green) and Nissl counterstaining (cyan). Scale bar, 100 μm. (**e**–**g**) Schematic diagrams of sagittal planes at 0.36, 0.60 and 0.84 mm interaural indicate the position of microscopic fields corresponding to confocal images (black square). Major neuronal tracts are schematized (colour code in Supplementary Fig. 3). Confocal images show the post-commissural axonal projections expressing GFP (green) in WT and MAP6-KO mice. In MAP6-KO mice, only some rare axons reached the mammillary body (**g**, arrows). Scale bars, 40 μm. Acb=accumbens nucleus; ac=anterior commissure; AHN=anterior hypothalamic nucleus; CA1=field Corn d'Ammon1 of the hippocampus; df=dorsal fornix; DS=dorsal subiculum; DG=dentate gyrus; HF=hippocampal formation; LS=lateral septal nucleus; mcht=medial corticohypothalamic tract; lfp=longitudinal fasciculus of the pons; MB=mammillary bodies; MM=medial mammillary nucleus medial part; pre-f=pre-commissural fornix; pf=post-commissural fornix; sm=stria medularis; Spt=septum; vhc=ventral hippocampal commissure.

The post-commissural fornix is the major efferent pathway of the hippocampal projection that originates from the subiculum^37^ and terminates at the posterior hypothalamus in the mammillary body (Fig. 3a). To characterize in greater detail the changes in the anatomy of the post-commissural fornix in adult, the *Thy1*-eYFP-H transgene^38^,^39^, which is expressed by subicular neurons, was introduced in MAP6-KO mice by breeding. On coronal sections of hippocampal formation including the dorsal subiculum (DS) from WT/ *Thy1*-eYFP-H and MAP6 KO/*Thy1*-eYFP-H adult mice, subicular neurons expressing eYFP were comparably distributed in the subiculum of both genotypes (Supplementary Fig. 2a,b) without change in density (Supplementary Fig. 2c). This indicates that subicular neurons, which project their axons in post-commissure, persist in MAP6-KO mice.

To visualize the entire fornix and analyze its axon bundling, we prepared sagittal slices with a cutting angle of 30° (Fig. 3b, coronal diagram). Panoramic views showed that the dorsal and the pre-commissural fornices, which contain fibres from Ammon's horn and septal part of the subiculum, are similar in WT and MAP6-KO mice (Fig. 3b, green framed panels). In contrast, the post-commissural fornix was shorter and thinner in MAP6-KO brain (Fig. 3b; green framed panels; observed in 3/3 MAP6-KO mice). Many MAP6-KO axons split unusually off from the fornix pathway and exhibit a chaotic organization with disordered and defasciculated axons as compared with WT (Fig. 3b, orange and blue framed panels). Accordingly, electron microscopy analysis of micro-dissected post-commissural fornices revealed clear axonal bundle defects in MAP6-KO brains (Fig. 3c). In MAP6-KO mice, most of the fornix fibres terminate scattered in the anterior hypothalamus, with only very few of them able to reach their final target, the mammillary body (Fig. 3b, yellow framed panels and Supplementary Fig. 2d–g).

We then examined the morphology of single axons from the fornix by performing stereotaxic injections of lentivirus expressing GFP into dorsal subiculum neurons (Fig. 3d). WT mice exhibit straight axonal projections with a smooth aspect, while MAP6-KO projections are abnormal, more tortuous and present many varicosities (Fig. 3e,f; observed in 3/3 MAP6 KO mice). Again, in MAP6-KO mice, only very few fibres reach the mammillary body (Fig. 3g).

Together these results demonstrate a clear neuronal disconnectivity in adult MAP6-KO mice brain, with the subiculum not properly connected to the hypothalamus.

### MAP6 regulates the embryonic development of the fornix

**Figure 4:**
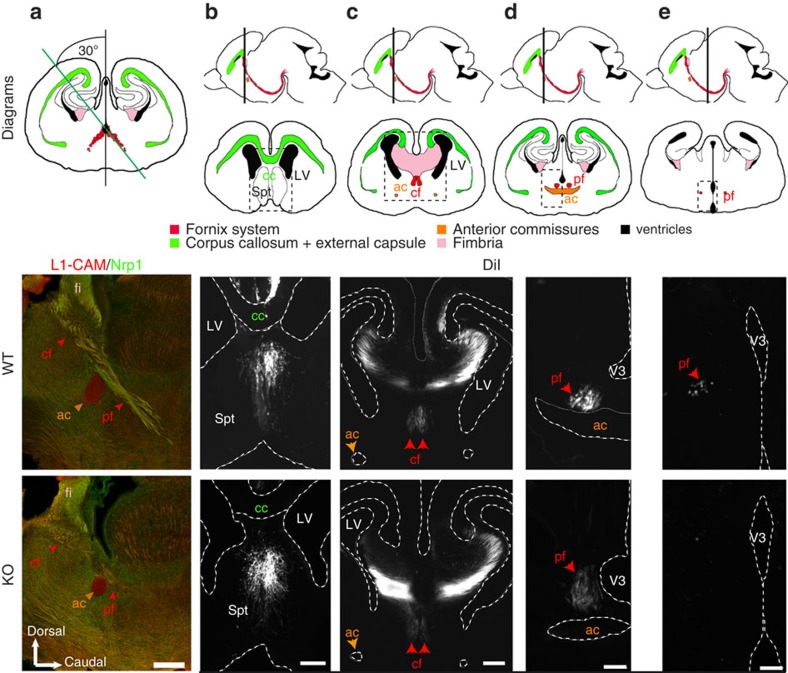
Absence of the post-commissural fornix in E18.5 MAP6-KO embryos. (**a**) Sagittal sections with a cutting angle of 30° of E18.5 WT and KO brain were double immunostained with anti L1 CAM and Neuropilin1 (Nrp1). A diagram shows the projection stack of post-commissural fornix in red on a coronal plan. The green line indicates the plane of the sagittal sections. Note that the size of the anterior commissure is strongly reduced and the post-commissural fornix is absent. Scale bars, 300 μm. (**b**–**e**) Coronal section of E18.5 WT and MAP6-KO brain after anterograde DiI tracing of the fornix from the subiculum. For each panel, a sagittal diagram shows the projection stack of post-commissural fornix in red and a grey line indicates the level of coronal plan. Dashed square indicates the position of microscopic field. Scale bars, **b**, **c** 100 μm; **d**,**e** 50 μm. ac=anterior commissure; cc=corpus callosum; cf=column of the fornix, fi=fimbria; LV=lateral ventricle; pf=post-commissural fornix; Spt=septum; V3=third ventricle.

We next asked whether defects in the descending post-commissural fornix reflect abnormal development or neurodegenerative processes. For this, we analysed the post-commissural fornix during neuro-development at embryonic day (E) 18.5 when the post-commissural fibres have reached their targets in the mammillary bodies. WT and MAP6-KO forebrains were double-immunostained with antibodies against the ubiquitous L1-CAM axonal marker and neuropilin1 (Nrp1), a semaphorin receptor enriched in subicular projections^36^. Results reveal that the post-commissural fornix is altered in KO embryos with a progressive reduction of axonal density from the column to the post-commissural part of the fornix and an absence of axonal projections after the anterior commissure (Fig. 4a and Supplementary Fig. 3; phenotype observed in 4/4 MAP6-KO mice and 0/4 WT mice). Similar results were obtained using DiI tracing of subicular neurons (Fig. 4b–e, phenotype observed in 2/2 MAP6 KO and 0/3 WT mice). Altogether, the post-commissural fornix does not form properly during neurodevelopment in the absence of MAP6 protein.

### MAP6 is involved in Sema3E- growth promoting activity

The absence of the postcommissural fornix in MAP6 KO is reminiscent of the phenotype of Sema3E-KO embryos^36^. Sema3E acts as an attractive and growth-promoting signal for axons of subicular neurons^36^. We therefore evaluated MAP6 function in the axonal response to Sema3E in cultured subicular neurons, which predominantly expressed the MAP6-E isoform (Supplementary Fig. 4a).

**Figure 5:**
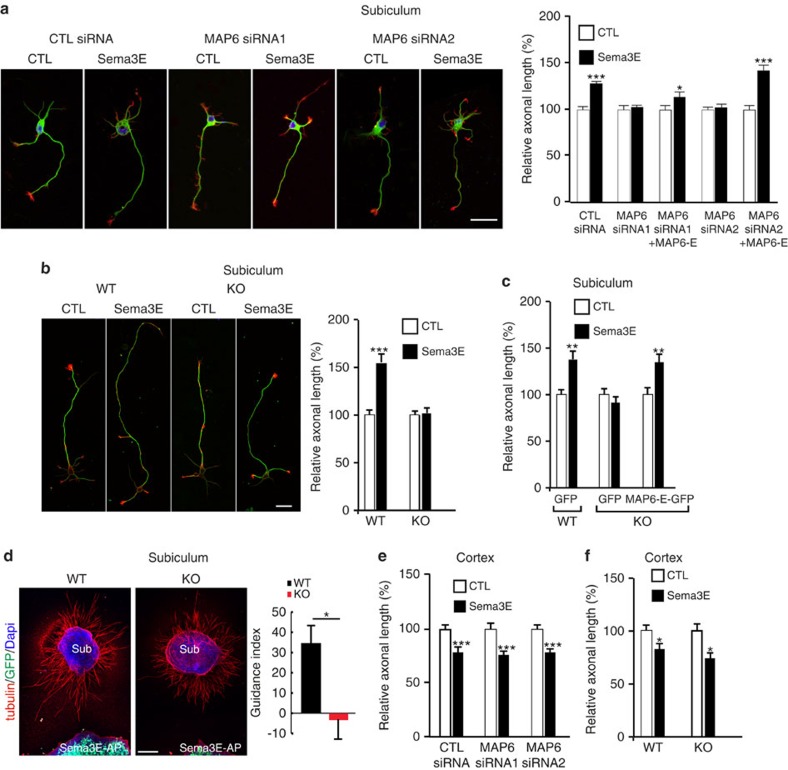
MAP6 is required for the growth-promoting activity of Sema3E on subicular neurons. (**a**) Typical images of subicular neurons cultured in the absence (CTL) or in the presence of 5 nM Sema3E for 24 h, electroporated with GFP-expressing vector and control siRNA or MAP6 siRNAs. To measure the axon length, neurons were stained with anti-α-tubulin (green) and phalloidin (red). Scale bar, 30 μm. The histogram shows quantifications of the relative axonal length in subicular neurons in the absence (CTL) or the presence of 5 nM recombinant Sema3E (Sema3E). Error bars, s.e.m., values corresponded to mean axonal length (measured in at least 60 neurons for each condition) and normalized to 100% for values obtained in control conditions. (**b**) Typical images of WT and MAP6-KO dissociated subicular neurons cultured in the absence (CTL) or the presence of 5 nM Sema3E. Scale bar, 30 μm. The histogram shows quantifications of relative axonal length of cultured subicular neurons in the absence (CTL) or the presence of 5 nM recombinant Sema3E. Error bars, s.e.m., values corresponded to mean axonal length (measured in at least 60 neurons for each condition) and normalized to 100% for values obtained in control conditions. (**c**) The histogram shows quantifications of relative axonal length after exogenous expression of rat MAP6-E-GFP. Error bars, s.e.m., values corresponded to mean axonal length (measured in at least 60 neurons for each condition) and normalized to 100% for values obtained in control conditions. (**d**) Sema3E axonal guidance response. Representative images of subiculum (Sub) explants from E17.5 WT or KO embryo co-cultured in a plasma clot with an aggregate of HEK cells transfected with Sema3E-AP and GFP. Scale bar, 200 μm. Quantification of the guidance response, graph shows mean guidance index (see methods) calculated for each condition (WT, *n*=11 and KO, *n*=8). Positive values indicate attraction. (**e**) Quantifications of axonal length in cultured cortical neurons in the absence or the presence of 5 nM recombinant Sema3E electroporated with GFP-expressing vector and control siRNA or MAP6 siRNAs. Error bars, s.e.m. Values corresponded to mean axonal length (measured in at least 60 neurons for each condtion) and normalized to 100% for values obtained in control conditions. (**f**) Quantifications of axonal length in cultured WT and MAP6-KO cortical neurons in the absence (CTL) or the presence of 5 nM recombinant Sema3E. Error bars, s.e.m., values corresponded to mean axonal length (measured in at least 60 neurons for each condition) and normalized to 100% for values obtained in control conditions. All quantifications have been repeated at least three times using independent neuronal cultures yielding consistent results. *P<0.05; **P<0.01; ***P<0.00; Student's *t*-test (5**a**,**b**,**c**,**e**,**f**); non-parametric Mann–Whitney *U*-test (5 **d**).

As previously reported^36^, application of Sema3E (5 nM) to WT cultured subicular neurons electroporated with siRNA control for 24 h, induced a 1.3-fold increase in axon length (Fig. 5a). When subicular neurons were treated with MAP6 siRNAs to knockdown MAP6 proteins (Supplementary Fig. 4b) they failed to respond to Sema3E (Fig. 5a). The co-electroporation of siRNAs and a cDNA coding for MAP6-E (resistant to siRNA) rescued the stimulation effect of Sema3E (Fig. 5a). Experiments performed with WT and MAP6-KO subicular neurons yielded to similar results with a 1.5-fold increase in the axon length in WT and no response in MAP6-KO neurons (Fig. 5b). The transfection of MAP6-E cDNA into MAP6-KO subicular neurons fully rescued the growth-promoting activity of the Sema3E (Fig. 5c). To determine whether MAP6 is involved in axonal guidance, explants of subiculum from WT and MAP6 KO brains were co-cultured with aggregates of HEK293T17 cells expressing Sema3E (Fig. 5d). Whereas WT subicular axons were attracted to Sema3E, MAP6-KO axons were unresponsive to this cue. These results clearly indicate that MAP6 protein contributes to the Sema3E-induced growth and guidance of subicular axons.

Sema3E is a bifunctional molecule, which in addition to its promoting effect on subicular axons can also inhibit growth of cortical axons^36^. However, despite a similar expression pattern of MAP6 isoforms between cultured subicular and cortical neurons (Supplementary Fig. 4a), the inhibition of MAP6 expression using siRNAs or the absence of MAP6 in KO neurons did not alter Sema3E responsiveness of cultured cortical neurons (Fig. 5e,f).

Together these results indicate a specific role for MAP6 in the growth promoting and attractive activity of Sema3E on subicular neurons and not in cortical neurons.

To estimate a possible *in vivo* genetic cooperation between MAP6 and Sema3E during fornix development, we performed analyses of trans-heterozygous animals. The fornix was analysed, from anterior commissure to mammilary bodies at P1. A strong delay of fornix formation was observed in double Sema3E/MAP6 heterozygous animals stronger than in single MAP6 or Sema3E heterozygous mice (Supplementary Fig. 5a–c). Furthermore, the defect in post commissural fornix persisted in P21 double heterozygous with a strong defasciculation (Supplementary Fig. 5d, arrows).

The fornix defects found in double heterozygous animals, but not in single heterozygous mice, are strong indicators of *in vivo* cooperation of MAP6 and Sema3E during fornix development.

### The Sema3E receptors are functional in MAP6-KO mice

**Figure 6:**
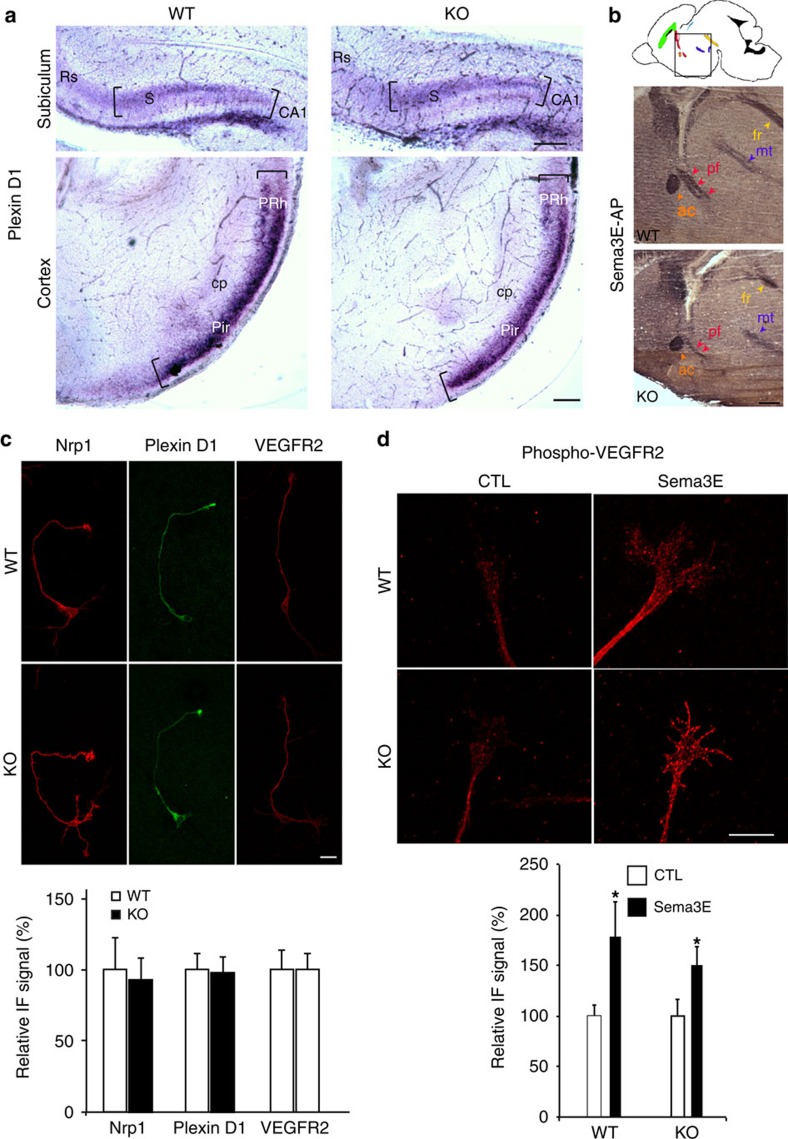
The Sema3E receptors (PlexinD1/Neuropilin1/VEGFR2) expression in developing MAP6-KO brain and in cultured subicular neurons. (**a**) Coronal brain slices of WT and MAP6-KO E17.5 embryos were hybridized with probes for PlxD1. PlxD1 mRNA is expressed in the subiculum and in the ventrolateral part of the cortex including the piriform cortex and the perirhinal cortex. Scale bar, 100 μm. S=subiculum; CA1=Ammon's Horn; cp=cerebral peduncle; Rs=retrosplenial cortex; Pir=piriform cortex; PRh=perirhinal cortex. (**b**) Binding of sema3E along the fornix. Schematic diagram of a sagittal section with major neuronal tracts indicated (colour code in supplementary Fig. 3). The square indicates the position of microscopic field shown below. The atrophic fornix post-commissural part (pf) observed in MAP6-KO embryos binds Sema3E-AP. (**c**) Immunolabelling of subicular neurons using anti-PlxD1, -Nrp1 and -VEGFR2 antibodies. Histogram shows quantification of the relative immunofluorescence signal. The values represent the mean pixel intensity of the staining (number of neurons≥60). Scale bar, 20 μm. (**d**) Immunolabelling of growth cones from subicular neurons using anti-phosphotyrosine antibody to Tyr1175 in VEGFR2 after 15 min of treatment with 10 nM of Sema3E. Histogram shows the mean pixel intensity of phospho-VEGFR2 staining relative to CTL conditions (number of growth cones≥20). Scale bar, 10 μm. Error bars, s.e.m. *P<0.05 Student's *t*-test.

To begin to understand the mechanism by which MAP6 controls axonal response to Sema3E, we examined the expression of the Sema3E receptor complex in MAP6-KO mice. Attractive effect of Sema3E on axonal growth depends on binding to a receptor complex composed of PlexinD1 (PlxD1), Neuropilin1 (Nrp1) and the vascular endothelial growth factor receptor 2 (VEGFR2); PlxD1 being the binding subunit and VEGFR2 the signal transducing subunit^35^. We determined PlxD1 receptor expression in the subiculum of WT and MAP6 KO E17.5 embryos by *in situ* hybridization and found no obvious differences (Fig. 6a). Accordingly, we observed a clear binding of Sema3E-alkaline phosphatase fusion protein (Sema3E-AP) to brain sections of WT and MAP6-KO embryos. Sema3E-AP labelled several axonal tracts, including anterior commissure, mammillo-thalamic tract and post commissural part of the fornix (Fig. 6b), although the staining was less intense in MAP6-KO brain slices due to the size reductions of axonal tracts. Thus, Sema3E can properly bind to its receptor in MAP6-KO brain. We then investigated the expression pattern of PlxD1, Nrp1 and VEGFR2 proteins, in MAP6-KO subicular neurons by quantitative immunofluorescence (Fig. 6c). In the absence of MAP6, PlxD1, Nrp1 and VEGFR2 expression levels were not altered (Fig. 6c).

In subicular neurons, Sema3E initiates intracellular signalling through VEGFR2, with phosphorylation of VEGFR2 tyrosine-1175 being an early event^35^. Using quantitative immunofluorescence, we found that Sema3E exposure increases VEGFR2 phosphorylation in both WT and MAP6-KO neurons (Fig. 6d). Therefore, the functional defect in MAP6-KO subicular neurons is likely to occur in post-receptor signalling.

### MAP6 interacts with components of the Sema3E receptor complex

**Figure 7:**
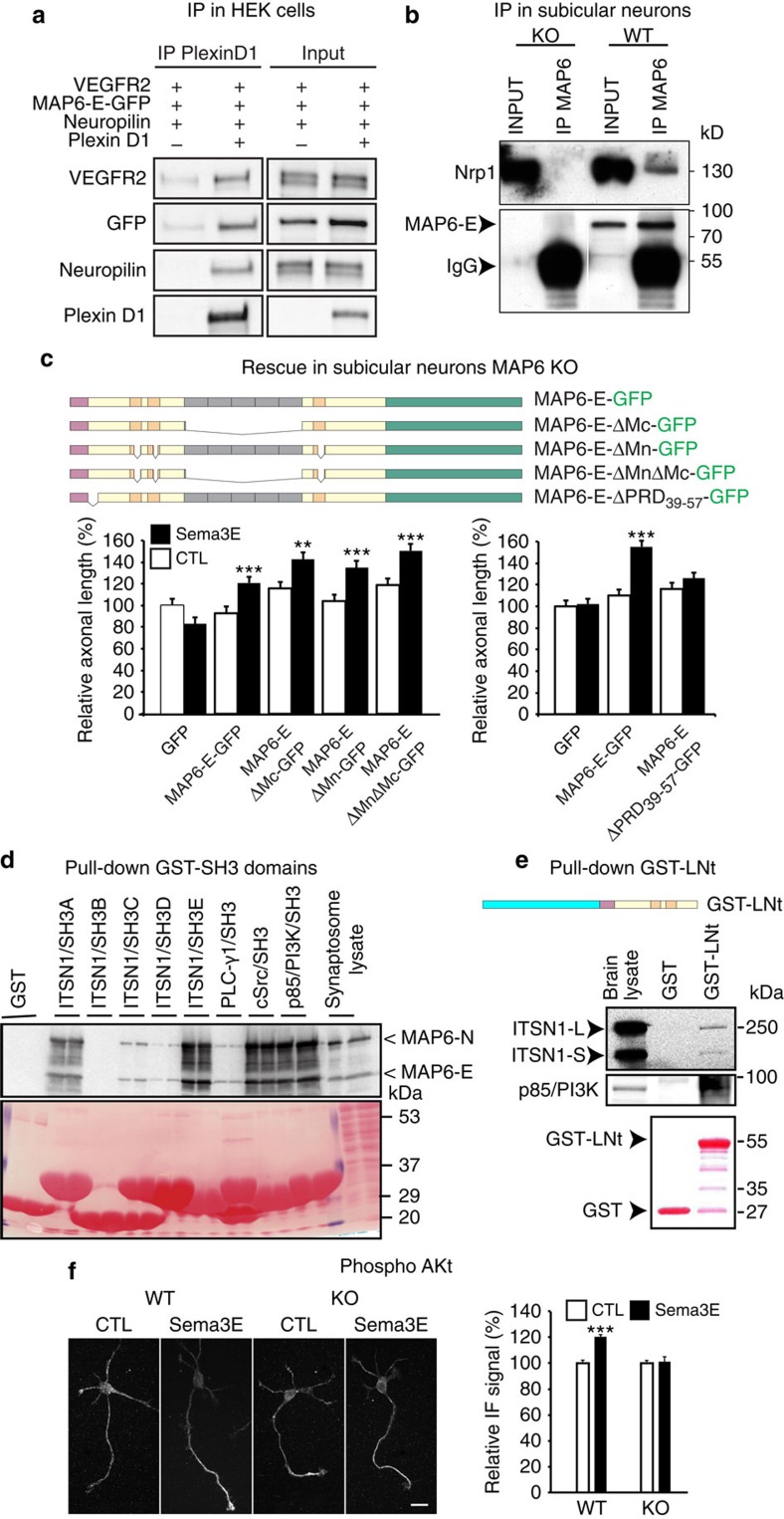
MAP6 protein is involved in the Sema3E signalling pathway. (**a**) Immunoprecipitation of MAP6-E-GFP and Sema3E receptor proteins. HEK 293T17 cells were transfected with MAP6–E-GFP, VEGFR2, Plexin D1 or Neuropilin1 cDNAs. Immunoprecipitation was performed using a polyclonal anti-PlxD1 antibody. In control experiments, Plexin D1 cDNA was omitted. (**b**) Immunoprecipitation of endogenous MAP6-E from subicular neurons using an anti-MAP6 23N antibody. (**c**) Rescue experiments on MAP6-KO neurons by MAP6-E-GFP constructs and Sema3E response. Schematic representation of MAP6-E-GFP, MAP6-E-ΔMnΔMc-GFP, MAP6-E-ΔMc-GFP, MAP6-E-ΔMn-GFP and MAP6-E-ΔPRD_39-57_-GFP constructs. Values correspond to mean axonal length and were normalized to 100% for values obtained in control conditions (GFP without Sema3E). All quantifications have been repeated at least three times using independent neuronal cultures yielding consistent results (number of neurons≥70 in all conditions). Error bars, s.e.m. **P<0.01; ***P<0.001 Student's *t*-test. (**d**) Immunoblot analysis of synaptosomal proteins eluted from glutathione-Sepharose columns containing GST or GST fused to SH3 domains from intersectin (ITSN1/SH3A-E domains), PLC-γ1, cSrc, and p85/PI3K. The blot was stained with Ponceau red and probed with 23N antibody. (**e**) Immunoblot analysis of postnatal brain proteins eluted from glutathione-Sepharose columns containing GST and GST-MAP6 LNt fragment containing the PRD_39-57_ domain. Blots were stained with Ponceau Red and probed with anti-p85 or anti-intersectin antibodies. (**f**) Immunolabelling, using anti-phospho-Akt antibodies, of subicular neurons treated 15 min with 10 nM of Sema3E. Histogram shows the relative immunofluorescence signal of phospho-Akt staining within axons and growth cones (number of neurons≥60). Error bars, s.e.m. ***P<0.001.

We next looked for a possible interaction of MAP6 with the Sema3E receptor complex. PlxD1, Nrp1 and VEGFR2 cDNAs were simultaneously expressed in HEK293T17 cells in combination with MAP6-E-GFP. In PlxD1 immunoprecipitates, we were able to detect Nrp1, VEGFR2 and MAP6-E-GFP proteins (Fig. 7a and Supplementary Fig. 6). Similar results were obtained when the immunoprecipitation was performed with an Nrp1 antibody (Supplementary Fig. 7a). In additional experiments PlxD1, Nrp1 and VEGFR2 cDNAs were individually expressed in HEK293T17 cells in combination with MAP6-E-GFP. We were able to detect MAP6 protein in PlxD1, Nrp1 and VEGFR2 immunoprecipitates (Supplementary Fig. 7b). We next determined whether the interaction of MAP6 with the Sema3E receptor occurs in cultured subicular neurons. Indeed, we were able to detect Nrp1 in MAP6 immunoprecipitates (Fig. 7b and Supplementary Fig. 7c). In control experiments using MAP6-KO neurons, Nrp1 was not detected. Together the data indicate that MAP6 is associated with the Sema3E tripartite receptor.

### MAP6 proline-rich domains and Sema3E promoting growth ability

To determine whether MAP6 participates in Sema3E signalling through its MT-binding inactivity we performed rescue experiments on cultured MAP6 KO subicular neurons using GFP-tagged MAP6-E mutants (Fig. 7c and Supplementary Fig. 8). As found previously (Fig. 5c), MAP6-E-GFP was able to rescue growth-promoting activity of Sema3E, whereas GFP control protein could not rescue (Fig. 7c). We then designed MAP6-E mutants devoid of MT-binding domains and evaluated their ability to rescue the growth-promoting activity of Sema3E. MAP6 proteins contain two types of MT-binding domains, the Mc modules in the central domain (in grey, Fig. 7c) and the Mn modules (in orange, Fig. 7c). As expected mutant lacking both sets of modules, MAP6-E-ΔMnΔMc-GFP, failed to bind MTs (Supplementary Fig. 8). However, this mutant rescued the growth-promoting activity of Sema3E (Fig. 7c). Similarly, MAP6-E lacking either MT-binding domain Mn or Mc (MAP6-E-ΔMn-GFP or MAP6-E-ΔMc-GFP) also rescued the growth-promoting activity of Sema3E (Fig. 7c). These results clearly indicate that MAP6 ability to bind MTs is not required for its role in Sema3E growth promotion.

### MAP6 interacts with SH3 domain containing proteins

Sema3E growth promotion involves VEGFR2 activation, which triggers the recruitment and the activation of SH2 and/or SH3 domain containing proteins such as PI3K^35^,^40^,^41^. In its N-terminal domain, MAP6-E contains stretches of proline residues, which are likely candidates for interaction with SH3-containing proteins, and it is known that MAP6 binds to the SH3-A domain of intersectin1^42^. We therefore deleted a proline-rich domain (PRD) of MAP6-E (MAP6-E-ΔPRD_39-57_). This mutant retains a MT-binding activity (Supplementary Fig. 8) but failed to rescue the growth-promoting activity of the Sema3E (Fig. 7c). Therefore, the ability of MAP6 to mediate Sema3E signalling via PlxD1/Nrp1/VEGFR2 requires the proline-rich domain (PRD_39-57_) and suggests that MAP6 interaction with SH3 domain-containing proteins is crucial for Sema3E growth-promoting activity.

We then performed a large screen to identify SH3 domain-containing proteins able to interact with MAP6 (Fig. 7d and data not shown). Lysates of isolated mouse brain were incubated with a variety of recombinant GST-SH3 protein domains. The two neuronal MAP6 isoforms (MAP6-E and MAP6-N) that both contain the PRD domain interacted with a broad range of SH3 domains to markedly varying extents. MAP6 strongly interacted with two SH3 domains of intersectin1 (ITSN1) and with the p85 subunit of PI3K (Fig. 7d), a protein involved in the neuronal VEGFR2 signalling pathway^35^.

We further analysed MAP6 interaction with intersectin1 and the p85 subunit of PI3K using the converse pull-down assays. A fragment of MAP6 protein corresponding to the N-terminal part of the protein (LNt fragment) that contains the proline-rich domain (PRD_39-57_) coupled to GST was produced. Analysis of pull-downs performed with brain lysates revealed that GST-LNt fragment associated with intersectin1 isoforms and with the p85 subunit of PI3K (Fig. 7e). Furthermore, as p85 subunit of PI3K is endogenously expressed in HEK 293T17 cells, we analysed its presence in MAP6 immunoprecipitates performed on HEK 293T17 cells and detected it as shown in Supplementary Fig. 7d. As the PIK3 effector, Akt is phosphorylated in the Sema3E pathway^35^, we then measured Akt phosphorylation following Sema3E exposure in WT and MAP6-KO subicular neurons. While Sema3E application increased the phosphorylation of Akt in WT neurons, it had no effect on MAP6-KO neurons (Fig. 7f). These results suggest that following Sema3E exposure, MAP6 might couple the Sema3E receptors to PI3K for activation of the Akt pathway.

## Discussion

Our study reveals a signal transduction role for MAP6, a protein previously known mainly for its MT stabilization ability, in promotion of axon growth in specific axon tracts in the developing brain. MAP6 associates with the Sema3E receptor complex and MAP6 PRD_39-57_ domain is crucial for Sema3E signalling. The ability of MAP6 to interact with multiple SH3 domain-containing proteins including PI3K and intersectin, known to be triggered in signalling cascades after Sema3E receptors activation, is most probably crucially involved in Sema3E signalling. This signalling role for MAP6 explains the specific phenotype observed in MAP6-KO mice. The mice did not exhibit generalized deficits expected to be associated with loss of MT stabilization, but rather they fail to develop a highly specific axonal tract involved in cognitive deficits due to the loss of MAP6's ability to transduce signals from the Sema3E receptors complex in subicular neurons. Our data potentially explain the cognitive defects in MAP6-KO mice and highlight the major role of this signalling pathway in a key element of brain development.

MAP6-KO mice displayed a large spectrum of behavioural and cognitive impairments associated with neurotransmission and synaptic alterations^23^,^28^,^30^,^43^, which have been shown to positively respond to long-term typical and atypical neuroleptic treatment^23^,^31^,^32^. These features are reminiscent of human psychiatric diseases and MAP6-KO mice have been proposed to be a useful animal model for some aspects of the pathophysiology of psychiatric diseases including schizophrenia. Before the present study, nothing was known about how MAP6 contributes to these key neurodevelopmental processes. By performing the first precise analysis of MAP6-KO mice brain anatomy, we showed that MAP6 deletion strongly reduced brain volume and is accompanied by ventricle enlargement. Alteration of brain size is well documented in human psychiatric disorders^1^,^2^. Our general DTI data showed that the reduction of brain volume is associated with white matter defect, affecting several axonal tracts within the brain. Similar white matter abnormalities and related abnormal connectivity are also found in some brain regions of schizophrenic or bipolar patients^3^,^4^,^5^,^6^. This suggests that the anatomical changes observed in MAP6-KO mice may account for their schizophrenia-like behavioural and cognitive impairments.

Images from DTI and histological analyses revealed key defects in neuronal connectivity in MAP6-KO mice. Indeed, some axonal tracks such as the anterior commissure, the internal capsule, the cerebral peduncle and the fornix are severely affected either in their size and/or in their microstructures as detected by FA changes. The fornix is mostly composed from the axons of subicular neurons and was the most deficient tract, with a quasi disappearance of the post-commissural part. The inability of subicular neurons to sense Sema3E signalling in the MAP6-KO mice is likely to be the main contributor to the posterior fornix defect. Indeed, subicular neurons failed to respond to Sema3E growth promoting activity and axonal guidance. Furthermore the double Sema3E/MAP6 heterozygous animals exhibited a delay in fornix formation and a strong defasciculation in the posterior part of the fornix in adult mice. The fornix defects in MAP6-KO brain are more severe than those observed in Sema3E-KO mice, which exhibit only latency in fornix formation associated with a defasciculation, strongly suggesting that additional molecular defects might be involved.

Our results indicate that MAP6 is not involved in the repulsive growth activity of Sema3E on cortical neurons; however, some cortical tracts were found abnormal with defasciculation (corpus callosum, internal capsule) indicating possible additional functions for MAP6.

Fornix disruption in MAP6-KO brains resulted in a disconnection between the hippocampus and the hypothalamus and this impaired connectivity most likely contributes to the observed behavioural disorders. The fornix belongs to the Papez circuit known to be involved in cognitive and memory abilities for review^44^. MRI and in particular DTI methods in humans report similar connectivity defects in schizophrenic patients with disrupted fornix integrity^45^,^46^,^47^,^48^,^49^ associated with alterations of resting state functional connectivity^50^ and defective memory organization^51^,^52^. Other links between abnormal neuronal axonal tract formation and specific genes have been reported in both animal models and patients. For example, defective schizophrenia-related genes such as *DISC1*^53^,^54^, *dysbindin*^55^,^56^ and *neuregulin*^57^ are linked to abnormal brain connectivity. Thus, abnormal tract formation and white matter defects, easily detectable by the DTI technique in humans, might be seen as a convergent consequence of a specific gene mutation in psychiatric disorders.

Sema3E binds to the PlexinD1 receptor both in cortical and subicular neurons, but signalling from here depends on a variety of co-receptor configurations. Thus, in cortical neurons PlexinD1 receptors act alone and promote growth cone collapse mainly through deactivation of R-Ras^58^. In contrast, Plexin D1 in subicular neurons is associated with both Neuropilin1 and VEGFR2, the signalling component being VEGFR2 rather than PlexinD1. MAP6-KO cortical neurons responded normally to Sema3E, whereas the subicular neurons did not respond, indicative of a defect in the VEGFR2 transducing pathway. We found that Sema3E is unable to trigger the PI3K/Akt pathway in MAP6-KO neurons, which is required for increased axonal growth. Our results suggest Sema3E binding to the extracellular domain of Plexin D1 allows the transactivation of VEGFR2 as indicated by its auto-phosphorylation, and the binding of MAP6 to the tripartite PlxD1/Nrp1/VEGFR2 receptor. Furthermore, MAP6, through a proline-rich domain, can recruit to the receptor some signalling SH3 domain-containing proteins, including the p85 subunit of PI3K, to facilitate lipid phosphorylation and consequent recruitment and activation of the Akt kinase signalling pathway for the promotion of axonal growth.

MAP6 protein contains several stretches of proline residues and we demonstrate the importance of the PRD_39-57_ domain in Sema3E signalling. We also showed that MAP6 is able to interact with several SH3 domains-containing proteins, including intersectin1 and p85 (PI3K). This ability of MAP6 to bind SH3 domains-containing proteins is shared with other structural MAPs. MAP2 and tau bind SH3 domain proteins such as Grb2, Fyn and cSrc^59^,^60^,^61^ but the physiological relevance of such interactions for neuronal function has never been clearly established. In the case of MAP6, we identify a physiological mechanism that involves the interaction between a structural MAP and SH3 domain signalling proteins. Indeed the PRD_39-57_ domain of MAP6 is required to allow Sema3E growth-promoting activity on subicular neurons and fornix formation. By interacting with the tripartite PlxD1/Nrp1/VEGFR2 receptor and SH3 domain proteins, MAP6 might couple receptor activation to downstream Akt signalling pathways, providing a potential molecular explanation for its novel role in development of the fornix.

The requirement of MAP6 protein for proper axonal extension of subicular neurons through the integration of guidance cues might be shared by other MAPs. Indeed some MAPs have been reported to influence axonal growth and axonal tracts formation such as doublecortin and MAP1B, which regulate hippocampal and corpus callosal development^62^,^63^, but the implication or not of their ability to bind MTs in these functions was not specifically analysed.

Overall, our study demonstrates a new axon guidance cue integration role for MAP6 in mediating signal transduction from a specific PlxD1/Nrp1/VEGFR2 receptor complex that mediates axonal growth in neurons possessing this unique receptor combination. MAP6 signalling is required for growth of a subset of brain neurons, notably the subicular axons, which in turn are required for wiring of the post-commissural part of the fornix during brain development. Together our data implicate MAP6 in neurodevelopment of specific brain axon tracts that are involved in cognitive abilities throughout life.

## Methods

### Animals

Homogeneous inbred C57BL6/129SvPas-F1 mice were obtained by crossing MAP6 pure heterozygote 129SvPas male or female mice with MAP6 pure heterozygote C57BL6 male or female mice. The heterogeneous non-inbred BALBc/129 SvPas-F2 mice were obtained first by crossing BALBc with pure heterozygote 129SvPas MAP6 mice, then by crossing heterozygote BALBc/129 SvPas-F1 offspring together. Th1-eYFP line H mice^38^ were obtained from Jackson Labs (B6.Cg-Tgn (Thy-YFP-H) 2Jrs) and crossed with pure heterozygote C57BL6 MAP6 mice to generate C57BL6/Thy1-eYFP MAP6 mice colony. These mice were then crossed with heterozygote 129SvPas MAP6 to generate WT/ and KO/Thy1-eYFP-H littermate mice in homogeneous inbred C57BL6/129 SvPas-F1. Double heterogeneous Sema3E/MAP6 mice were obtained either by the crossing of C57BL6 Sema3E^+/−^ and 129vPas MAP6^+/−^ mice or C57BL6 Sema3E^+/−^ and C57BL6/Thy1-eYFP MAP6^+/−^ mice. Male and/or females mice were used between 2-4 months old. In accordance with the policy of the Institut des Neurosciences of Grenoble (GIN) and the French legislation, experiments were done in compliance with the European Community Council Directive of 24 November, 1986 (86/609/EEC). The research involving animals was authorized by the Direction Départementale de la protection des populations—Préfecture de l'Isère-France (Deloulme J. C., PhD, permit number 380822) and by the ethics committee of GIN n 004 accredited by the French Ministry for of Research.

### Magnetic resonance imaging

MRI was performed at 7T Bruker Biospec Avance III using a volume transmit/surface receive coil combination. The anaesthetized animal (1.5% isoflurane in air) was placed in heated cradle whose temperature was regulated at 37 °C. T2-weighted spin-echo images (TR/TE=5,000/42 ms, field of view=25 × 25 mm^2^, slice thickness=0.6 mm, 29 slices) were obtained across the entire brain (from the cerebellum to olfactory bulb). The scan duration was 11 min/animal. Subsequently, the following contours were manually delineated on the T2-weighted images: brain, ventricles.

### Diffusion tensor imaging

DTI experiments were carried out with the same MRI equipment. The DTI technique is based on water diffusion measurements in different spatial directions to assess the medium anisotropy. The FA parameter is derived from DTI measurements and describes voxel by voxel, the rate of anisotropy of water mobility that can be free or restricted in a given spatial direction. In GM and ventricles, water molecules diffuse without preferential direction and the FA value tends toward 0 while in white matter the FA value tends towards 1 describing a high medium anisotropy. Indeed water diffusion is free along fibre bundles (maximal diffusivity) and restricted perpendicularly (minimal diffusivity).

Technically, adult WT and MAP6-KO mice were perfused transcardially with saline followed by 4% paraformaldehyde (PFA) and imaged in an animal bed with ear bars and bite bar head fixation. *Ex vivo* diffusion-weighted images were acquired with a spin echo sequence in which two identical diffusion gradients were applied with a duration of Δ=4 ms, and a separation time Δ=10 ms. The repetition time and echo time were set to 2,000 and 35 ms, respectively. Six diffusion-weighted images were acquired with a b-value of 1,000 s mm^2^ and applied along six different orientations: [110], [1–10], [011], [01-1], [101] and [-101]^64^. The reference image was acquired with b around 50 s mm^2^. All images were acquired in a field of view of 15 mm × 15 mm and a matrix of 256 × 256 leading to an in-plane spatial resolution of 59 μm × 59 μm. Seven contiguous coronal slices of 0.7-mm thickness positioned between −3.2 and 2.7 mm of the Bregma were acquired. Twelve signal averages were used for a total imaging time of ≈12 h. All DTI maps pixels by pixels (diffusivity, FA, colour maps, FA-weighted colour maps) were obtained using in house software written in Matlab (The MathWorks, Natick, MA). The ROIs (regions of interest) were carefully delineated on FA-weighted colour map on the basis of histology images guided by the paxinos atlas of mouse brain^65^.

### Brain histology

Brains from adult C57BL6/129SvPas and C57BL6/129SvPas/Thy1-eYFP mice were fixed by transcardiac perfusion of a 4% PFA solution. Brains were extracted and stored in the same fixative solution at 4 °C. Brains were sliced at 30 μm using vibratom (Leica, VT1000S) and mounted on Superfrost plus slides (Menzel-Gläser). Coronal sections and transversal sections with a cutting angle of 30° from C57BL6/129SvPas/Thy1-eYFP mice were stained with Nissl Neurotrace stain (Invitrogen) and observed with stereoscopic microscope (Nikon, AZ100M) and with confocal microscope (Zeiss, LSM 710). Coronal sections of C57BL6/129SvPas mice were stained using the gold method^66^ and counterstained with cresyl violet. For the evaluation of subicular neuronal density, coronal section of Thy1-eYFP mice was immunostained with an NeuN antibody and eYFP-positive cells were quantified using an homemade ImageJ macro. In brief the dorsal subiculum ROI was drawn using the NeuN staining image and the eYFP local maxima intensity signal was detected after application of a bandpass filter. All the neuroanatomical abbreviations in this study were from ref. 65 except for pre-f, precommissural fornix; pf, postcommissural fornix, cf, column of the fornix; PVH, paraventricular hypothalamic nucleus; AHN, anterior hypothalamic nucleus. All morphometric measures were performed using ImageJ software.

Immunohistofluorescence on E18.5 using L1-CAM (Millipore; 1:2,000) and Nrp1 (R&D system; 1:200) and anterograde DiI tracing of the fornix were carried out with small crystals of lipophilic tracer DiI placed in the dorsal hippocampus.

### Lentiviral axonal tracing

Lentiviral vectors encoding the green fluorescent protein (GFP) was produced in 293T cells by transient calcium phosphate precipitation of a 4-plasmid system (packaging construct pCMVDR-8.92; pRSV-Rev plasmid, plasmid encoding the VSV-G envelop pMD.G; SIN-W-PGK-GFP transfer vector). In brief, the VSV-G pseudo-typed HIV-1 vector was concentrated by ultra-centrifugation and resuspended in PBS with 1% bovine serum albumin. The viral particle content was determined by p24 antigen enzyme-linked immunosorbent assay (RETROtek; Gentaur, Paris, France). The batches were stored at −80 °C until use. Concentrated viral batch were thawed on ice and resuspended by vortexing and repeated pipetting and diluted to a concentration of 490 ng p24 antigen/μl. Mice received a total volume of 0.75 μl of the vector preparation, administered at a rate of 0.2 μl min^1^, injected stereotaxically into the dorsal subiculum of adult WT and KO mice at the following coordinates (mm) anteroposterior (AP), mediolateral (ML) and dorsoventral (DV) relative to Bregma AP/ML/DV: −3.3/1.55/−1.8 and 3.2/1.5/−1.65, respectively. After 2 weeks, brains were removed after intracardial perfusion of 4% PFA, sliced at 30 μm using vibratom (Leica, VT1000S) and mounted on Superfrost plus slides (Menzel-Gläser). Saggital sections were stained with Nissl Neurotrace stain (Life Technologies) and observed with a confocal microscope (Zeiss, LSM 710).

### Plasmids and expression of recombinant proteins

MAP6-E corresponds to amino acids (aa) 1–614 of NP_058900. EGFP-tagged MAP6-E constructs were fused to a C-terminal EGFP, using plasmid pEGFP-N1 (Clontech), with the following modifications: ΔMn=deletion of aa 124–138+aa 161–171+aa 527–541; ΔMc=deletion of aa 226–451; ΔPRD39-57=deletion of aa 39–57; ΔMn3=deletion of aa 527–541. LNt refers to aa 1–225. GST-LNt refers to LNt fused to a N-terminal GST, using plasmid pGEX-6P-1 (GE Life Sciences). GST-tagged intersectin-SH3 A-E constructs were kindly provided by Peter McPherson (McGill University, Canada). GST-tagged cSrc-SH3 (aa 81–142, rat sequence) was subcloned to pGEX-4T-1. GST-tagged p85-SH3 (aa 1–79, mouse sequence) was subcloned into a pGEX-2T vector. GST-tagged PLCγ-SH3 (aa 790–852, rat sequence) was subcloned into pGEX-6P-1. GST constructs were transformed into *Escherichia coli* strain JM109 by heat shock and the expressed recombinant proteins were bound to glutathione (GSH)-sepharose beads according to the manufacturer's instructions (GE Healthcare).

### Electron microscopy

Mice were fixed by perfusion and fornix as well as corpus callosum were dissected before being quickly immersed in fixative (2% PFA and 2% glutaraldehyde in 0.1 M cacodylate buffer). Samples were then post-fixed with 1% osmium tetroxide, stained in 1% uranyl acetate and dehydrated in ethanol before being embedded in epoxy resin (Fluka). Ultrathin sections of the sample (60 nm) were stained with uranyl acetate and lead citrate before being viewed under a transmission electron microscope at 80 kV (JEOL 1200EX). Images were taken with a digital camera (Veleta, Olympus) and morphometric measurements were done with iTEM software (Soft Imaging System).

### Production and binding of Sema3E-AP, *in situ* hybridization

HEK293T cells were transfected with the Sema3E-AP vector or empty pAPtag-5 vector as control, using a calcium phosphate kit (LifeTechnologies). After 3 days of culture in Opti-MEM serum-free medium (Gibco), the supernatant was collected and concentrated using Centricon filters (Millipore). AP activity was measured using SIGMAFAST^™^ p-nitrophenyl phosphate tablets (Sigma Aldrich), as per the manufacturer's instructions. Sema3E-AP binding experiments on tissue sections and *in situ* hybridization were performed on 20 mm cryostat sections from WT and MAP6 KO E17.5 embryos^36^. PlexinD1 probe was generously provided by Mark Tessier-Lavigne.

### Neuron analysis

Brains from WT and KO MAP6 littermate embryos (E17.5–18.5) were dissected to the ventrolateral cortex and the subiculum. Tissue pieces were dissociated and plated onto polylysine/laminin-coated four-well plates (Nunc) in Neurobasal medium supplemented with 1 mM glutamine, 1:50 B27 (GIBCO), and control AP or Sema3E-AP supernatants. In some experiments, neurons were electroporated with a GFP-pCAGGS vector alone or together with MAP6-E cDNA or different siRNAs (100 pmol). Efficient knockdown of MAP6 was obtained using Stealth RNAi (Invitrogen), siRNA1 sequence 5′-GCG AGU ACA GUA AGC AGC UCU UAC A-3′ (siRNA1) and 5′-CCA AUA AGC CCA GUG CAG CGG ACA A-3′ (siRNA2). In some experiments, neurons were electroporated with control GFP and GFP-MAP6-E full-lenght or GFP-MAP6 deletion mutants. After 24 h *in vitro*, cultured cells were fixed and immunostained with an anti-tubulin antibody^67^ and labelled with Texas Red-X Phalloidin (1:1,000, Invitrogen). Quantification of axonal growth on cortical and subicular neurons in the presence or in the absence of Sema3E was performed by morphometric measures obtained using ImageJ software. Rescue experiments were obtained after transfection of MAP6 constructs in subicular neurons at plating using Amaxa transfections. Quantification of receptors and signalling protein expression was performed using anti-Neuropilin, goat anti Plexin D1, goat anti-VEGFR2 (R&D Systems) and anti-phosphoY1175-VEGFR2, anti-phosphoS473-Akt (Cell Signaling Technology).

### Co-culture experiments

To produce aggregates of Sema3E-expressing cells, HEK293T cells were trypsinized 4–6 h after transfection with the AP-Sema3E vector and resuspended in 0.2 ml of culture medium (DMEM-GlutaMAX (Gibco), heat-inactivated fetal bovine serum (1:10), 1 mM Sodium pyruvate, 100 U ml^−1^ Penicillin–streptomycin) per 60-mm well. Drops of the cell suspension were placed onto the lids of 35 mm dishes, which were inverted and incubated over culture medium for 16 h. Clusters of AP-Sema3E-expressing Hek293T cells were placed on glass coverslips together with explants of subiculum from E17.5 WT or MAP6-KO mouse embryos, and embedded in 15 μl of chicken plasma (Sigma) coagulated with 15 μl of thrombin (0.2 mg ml^−1^, 20 NIHU ml^−1^ Sigma). The co-cultures were incubated in neurobasal medium supplemented with 1 mM glutamine, B27 (GIBCO) (1:50) for 2 days at 37 °C, 5% CO_2_ in a humidified incubator. After fixation in 4% PFA, axons were immunostained with mouse anti-tubulin antibody (Sigma T9026, 1:1,000) and donkey anti-mouse Alexa Fluor 568 secondary antibody (Life Technologies A10037, 1:500). The guidance effect was quantified by calculating the P/D Ratio, where P and D represent the length of axons extending in the quadrants proximal and distal to the HEK 293T cell aggregate, respectively. Therefore, a P/D ratio of 1 indicates no bias towards or away from the source of Sema3E.

### Biochemical experiments

For Immunoprecipitation experiments, HEK-293 T17 (ATCC) cells were transfected with VEGFR2 and/or PlexinD1 and/or Neuropilin1^35^ and/or MAP6-E-GFP cDNAs using a calcium phosphate kit (LifeTechnologies). One day after transfection, cells were scraped with lysis buffer (20 mM Tris, 1 mM EGTA, 1 mM EDTA, 5 mM NaF, 1 mM DTT, 0.27 M sucrose, 0.5% Triton X-100, pH 7.2) in the presence of protease inhibitors (Complete Cocktail tablets, Roche). After centrifugation of the cell lysate at 12,000 *g* for 20 min at 4 °C, the supernatant was diluted 3 × with buffer C (2% glycerol, 10 mM Tris-HCL pH 7.5) and incubated for 4 h at RT with 20 μg Rabbit anti-MAP6 23N^25^ or 10 μg Goat anti PlexinD1 (R&D Systems) or 10 μg Goat anti Neuropilin (R&D Systems) or 10 μl Rabbit anti VEGFR2 (Cell Signalling). Complexes were precipitated by 35 μl Dynabeads Protein G- (Life Technologies) for 40 min at RT. The immunoprecipitates were washed successively with buffer A (0.2% NP-40, 150 mM NaCl, 2 mM EDTA, 2% glycerol, 10 mM Tris-HCL pH 7.5) buffer B (0.2% NP-40, 500 mM NaCl, 2 mM EDTA, 2% glycerol, 10 mM Tris-HCL pH 7.5) and buffer C. Complexes were separated by electrophoresis, blotted and revealed using the following antibodies: Rabbit anti-VEGFR2 (1/4,000, Cell Signaling), goat anti-PlexinD1 (1/1,000, R&D Systems), goat anti-Neuropilin (1/1,000, R&D Systems), rabbit anti-GFP (1/1,000 Life Technologies). Similar immunoprecipitates protocol was performed using subicular neurons grown for 3 days *in vitro* and using 23N rabbit polyclonal antibody against MAP6^68^. For pull-down experiments, brain lysates were incubated with the GSH-sepharose beads (GE Healthcare) coated with GST recombinant proteins and bound proteins analysed by western blot.

### Statistical analysis

Statistics were performed using the Prism 5.0 software (GraphPad Software) using tests as indicated in each figure.

## Additional information

**How to cite this article:** Deloulme, J. C. *et al*. Microtubule-associated protein 6 mediates neuronal connectivity through Semaphorin 3E-dependent signalling for axonal growth. *Nat. Commun*. 6:7246 doi: 10.1038/ncomms8246 (2015).

## Supplementary Material

Supplementary Information Supplementary Figures 1-8

## Figures and Tables

**Table 1:** FA values and ROI surface are expressed as mean±s.e.m.
