# Supplementary material for: Microtubule-associated protein 6 mediates neuronal connectivity through Semaphorin 3E-dependent signalling for axonal growth
Source: Nat Commun. 2015 Jun 3;6:7246. doi: 10.1038/ncomms8246 (PMC4468860; doi:10.1038/ncomms8246)
Supplement: Supplementary Information — Supplementary Figures 1-8 [file ncomms8246-s1.pdf]

Supplementary Figure 1. The integrity of myelin sheaths is not affected in MAP6 KO mice

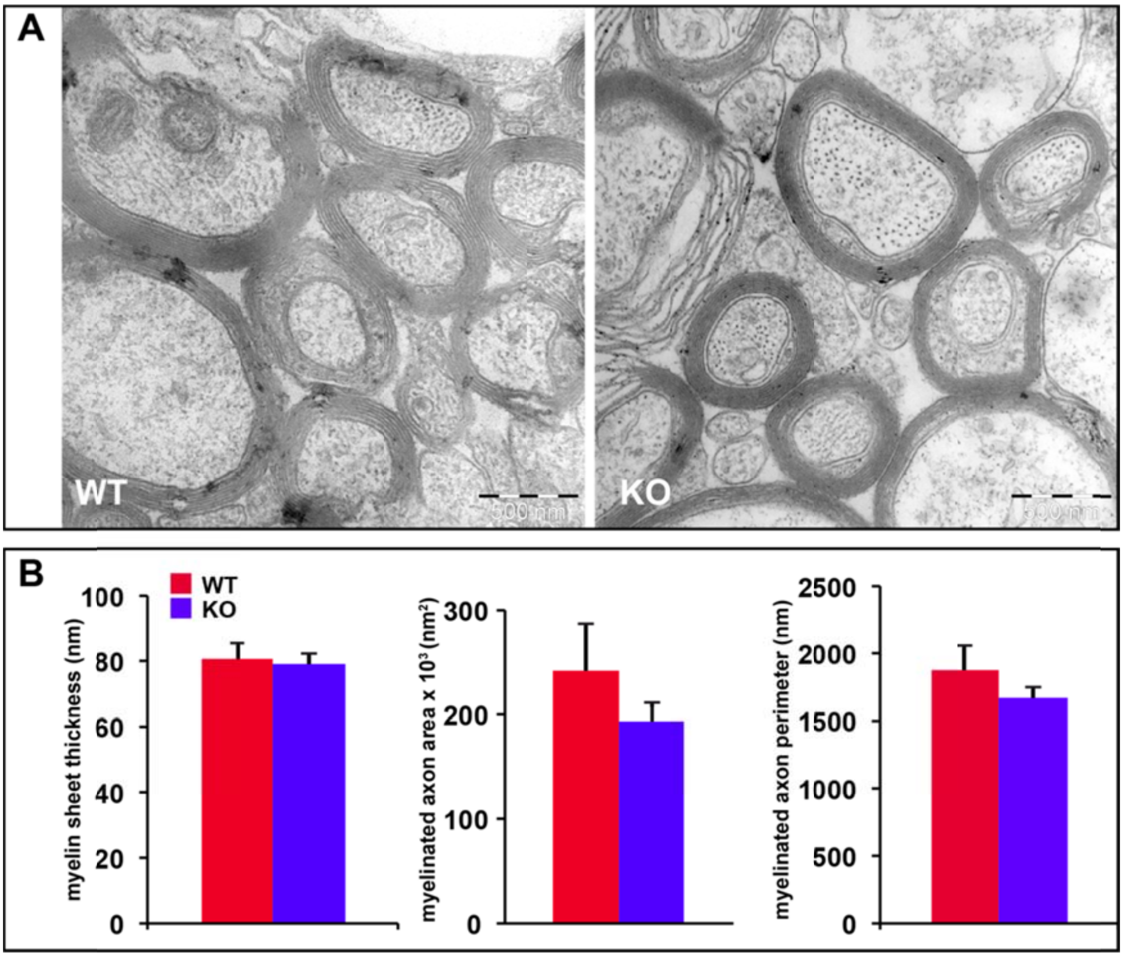

(A) Representative ultrastructural images of cross-sectioned axons within the corpus callosum from WT and KO mice.

(B) Quantifications of myelin sheath thickness, axon area and axon perimeter. Values are given as means ± SEM for five animals per genotype (n ≥ 40 axons measured per animal). No significant statistical differences were found between WT and KO using the Student's t test. Scale bar: 500 nm.

Supplementary Figure 2. MAP6 deletion results in the absence of the post-commissural fornix without obvious modification in subicular neuronal population

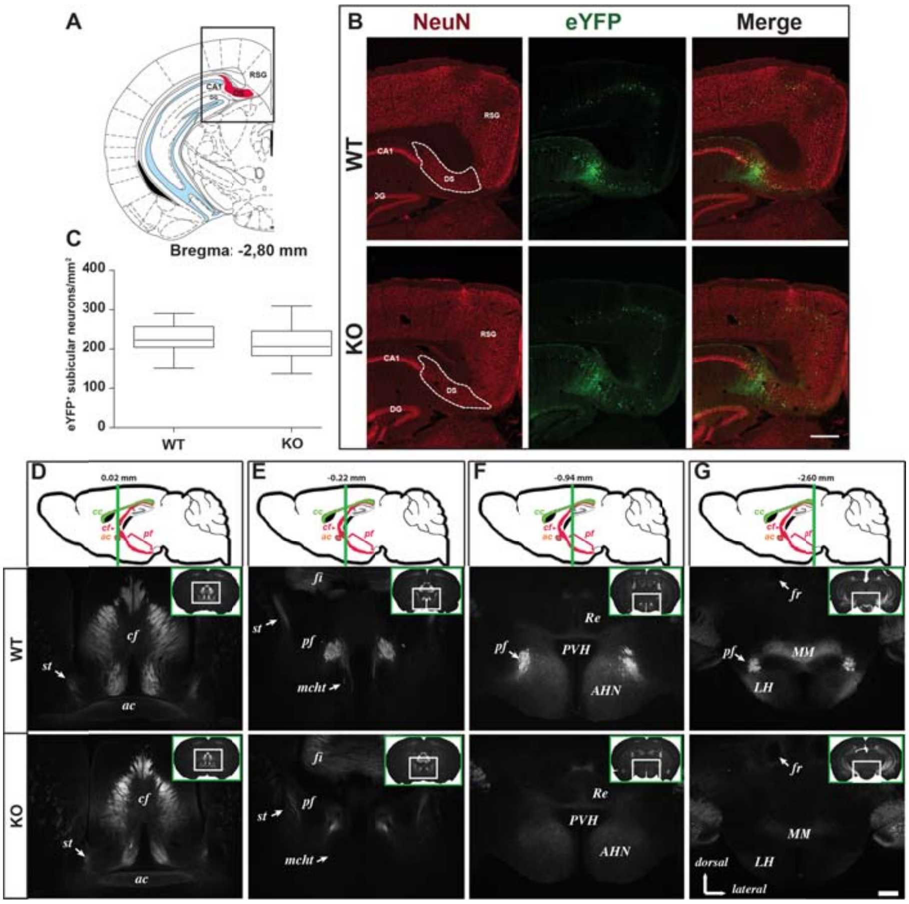

(A-C) Coronal sections of hippocampal formation from adult WT/Thy-eYFP-H and MAP6 KO/Thy-eYFP-H. The dorsal subiculum (DS) shown in the diagram of Bregma -2.80mm section (A) was delimited on NeuN immunolabeling slice (B, dashed ROI). The density of eYFP subicular neurons in WT and MAP6 KO dorsal subiculum was quantified and the results shown in (C). No significant difference was observed between genotype (n=5 animals for each genotype). Scale bar 400µm. (D-G) Fluorescence in WT/Thy-eYFP-H and MAP6 KO/ Thy-eYFP-H mice for 4 identical coronal sections. In the sagittal diagrams, the fornix and the post-commissural fornix are indicated in red. The green line indicates the coronal plane (Bregma value in mm). The position of microscopic field appears in insets. Scale bar 200µm.

Abbreviations: P, presubiculum; S, subiculum; DS, dorsal subiculum; cc, corpus callosum; DG, dentate gyrus; CA1, ammon's horn 1; cf, column of the fornix; pf, post-commissural fornix; ac, anterior commissure; st, stria terminalis; fi, fimbria; mcht, medial corticohypothalamic tract; Re, reuniens thalamic nucleus; PVH, paraventricular hypothalamic nucleus; AHN, anterior hypothalamic nucleus; fr, fasciculus retroflexus; MM, medial mammillary nucleus medial part; LH, lateral hypothalamic area.

Supplementary Figure 3: MAP6 is necessary for the development of the post-commissural fornix.

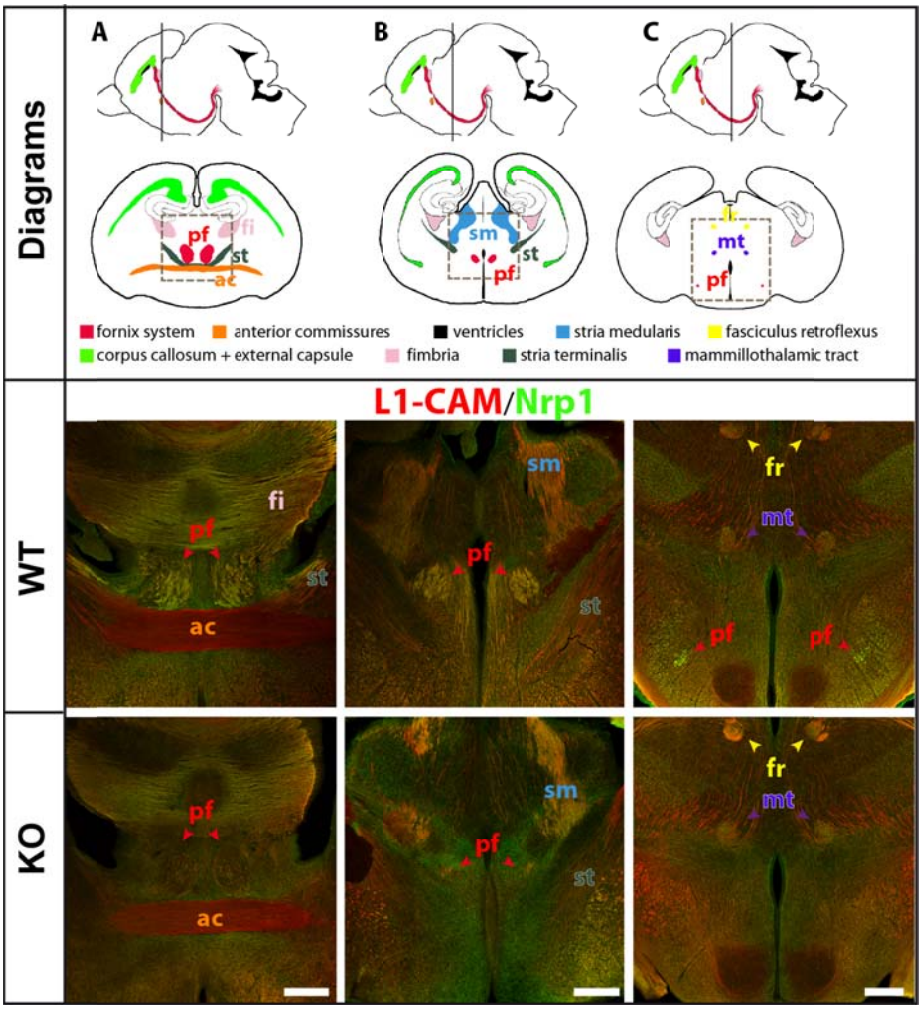

(A-C) Coronal sections of E18.5 WT and MAP6 KO brain double-immunostained with anti L1 CAM and neuropilin1 (Nrp1). For each panel, a sagittal diagram shows the projection stack of post-commissural fornix in red and a grey line indicates the level of coronal section. The position of the major neuronal tracts was indicated on a coronal diagram and the dashed square indicates the position of microscopic field. The fornix is indicated in red. Scale bars, 300 µm.

Abbreviations: cf, column of the fornix, pf, post-commissural fornix; .ac, anterior commissure; fi, fimbria; sm, stria medularis; st, stria terminalis; mt, mammillary tract; fr, fasciculus retroflexus.

Supplementary Figure 4. Expression of MAP6 proteins.

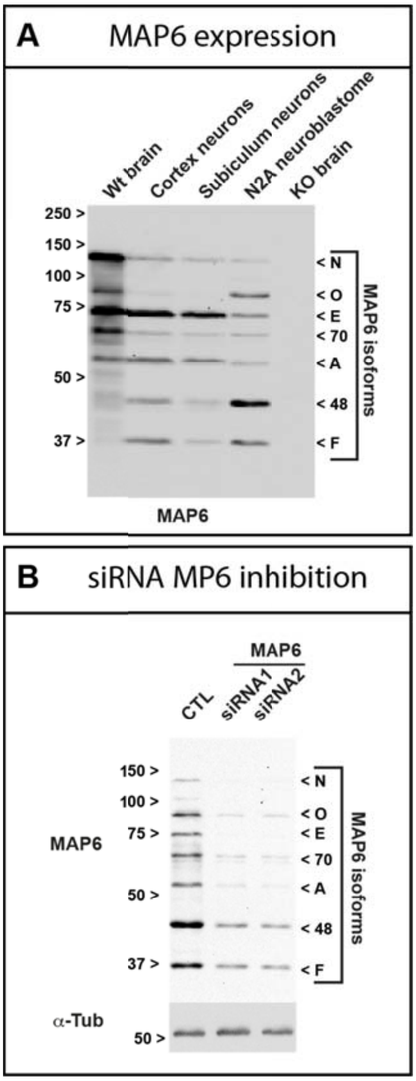

(A) Western blot of total protein from mouse brain homogenate (WT brain and MAP6 KO brain), cell extracts from mouse neuroblastoma Neuro-2A (N2A) and *in vitro* cultured subicular and cortical neurons (3 days of culture), blotted with MAP6 23N antibody.

(B) Western blot of crude extracts of cultured N2A cells treated for 48 h with MAP6 siRNA1, MAP6 siRNA2 and negative control siRNA (CTL), blotted with MAP6 23N antibody and with α-tubulin antibody (α-Tub). Both siRNAs induce a strong reduction of MAP6-E expression.

Supplementary Figure 5: MAP6 and Sema3E cooperate *in vivo* during fornix formation.

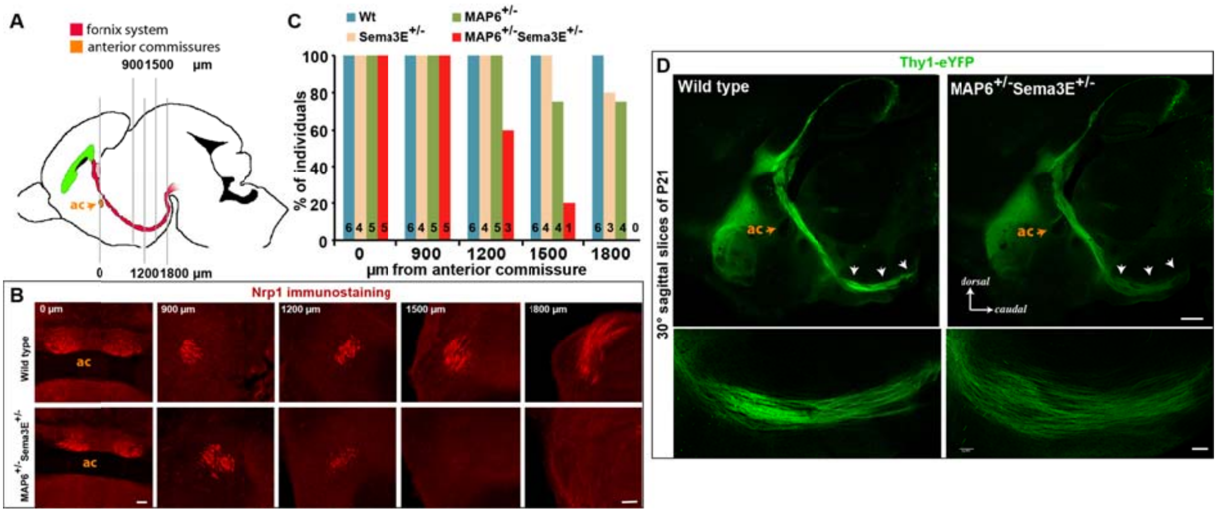

(A) Sagittal diagram showing the projection stack of post-commissural fornix in red. Grey lines indicate the level of coronal section through the fornix to the hypothalamus of P1 from WT, Sema3E heterozygous (Sema3E$^{+/-}$), MAP heterozygous (MAP6$^{+/-}$) and double Sema3E/MAP6 heterozygous (MAP6$^{+/-}$ Sema3E$^{+/-}$) mice.

(B) Brains coronal sections after immunolabelling with anti Nrp1 antibody. As compared to WT, the formation of the fornix is strongly delayed in double heterozygous MAP6$^{+/-}$ Sema3E$^{+/-}$ animals. Scale bars, 100 µm.

(C) Quantifications of the caudal extension of the post-commissural fornix from the anterior commissure (0µm) to the mammilary body (+1800 µm), for each genotype. In WT the fornix is visible in all sections (6/6 animals). In Sema3E$^{+/-}$/MAP6$^{+/-}$ animals the fornix was visible at 900 µM from the anterior commissural in 5/5 animals but only in 1/5 animal at 1500µm and in 0/5 animal at 1800µM. A milder phenotype was observed for single heterozygous MAP6$^{+/-}$ and Sema3E$^{+/-}$ animals; the fornix was visible on 3/4 and 4/5 at +1800µm.

(D) Analysis of the fornix using WT/Thy1-eYFP-H and MAP6$^{+/-}$/Sema3E$^{+/-}$/Thy1-eYFP-H P21 mice in sagittal slices with a cutting angle of 30°. In double heterozygous MAP6$^{+/-}$ Sema3E$^{+/-}$ brains, the fornix is strongly defasciculed in the terminal part of the post-commissural fornix (arrows). Scale bars, 500 µm.

Supplementary Figure 6: Full blots from main Figure 7A

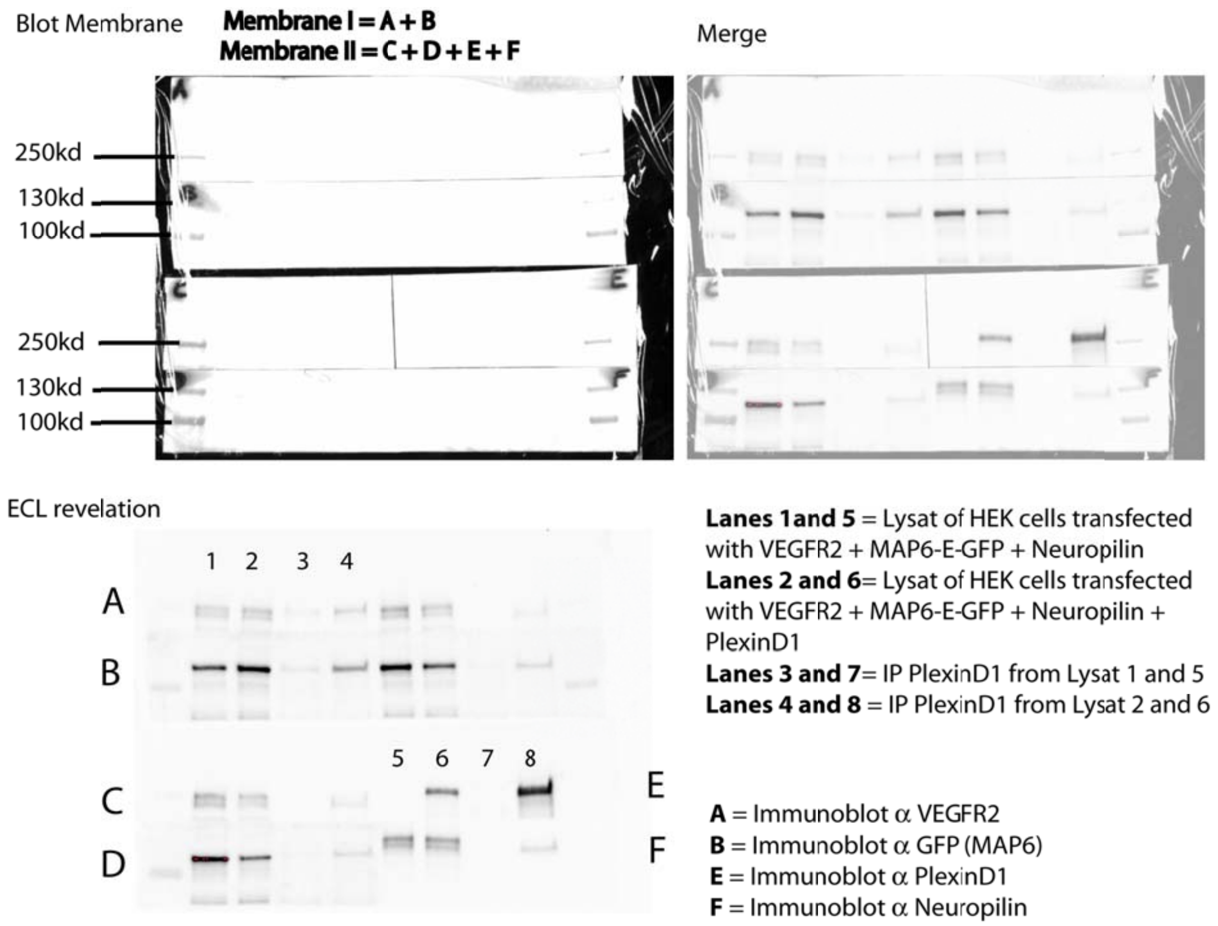

Bands from ECL revelation in numbered lanes correspond to Figure 7A

Supplementary Figure 7: MAP6 interacts with Sema3E tripartite receptor components and p85/PI3 kinase

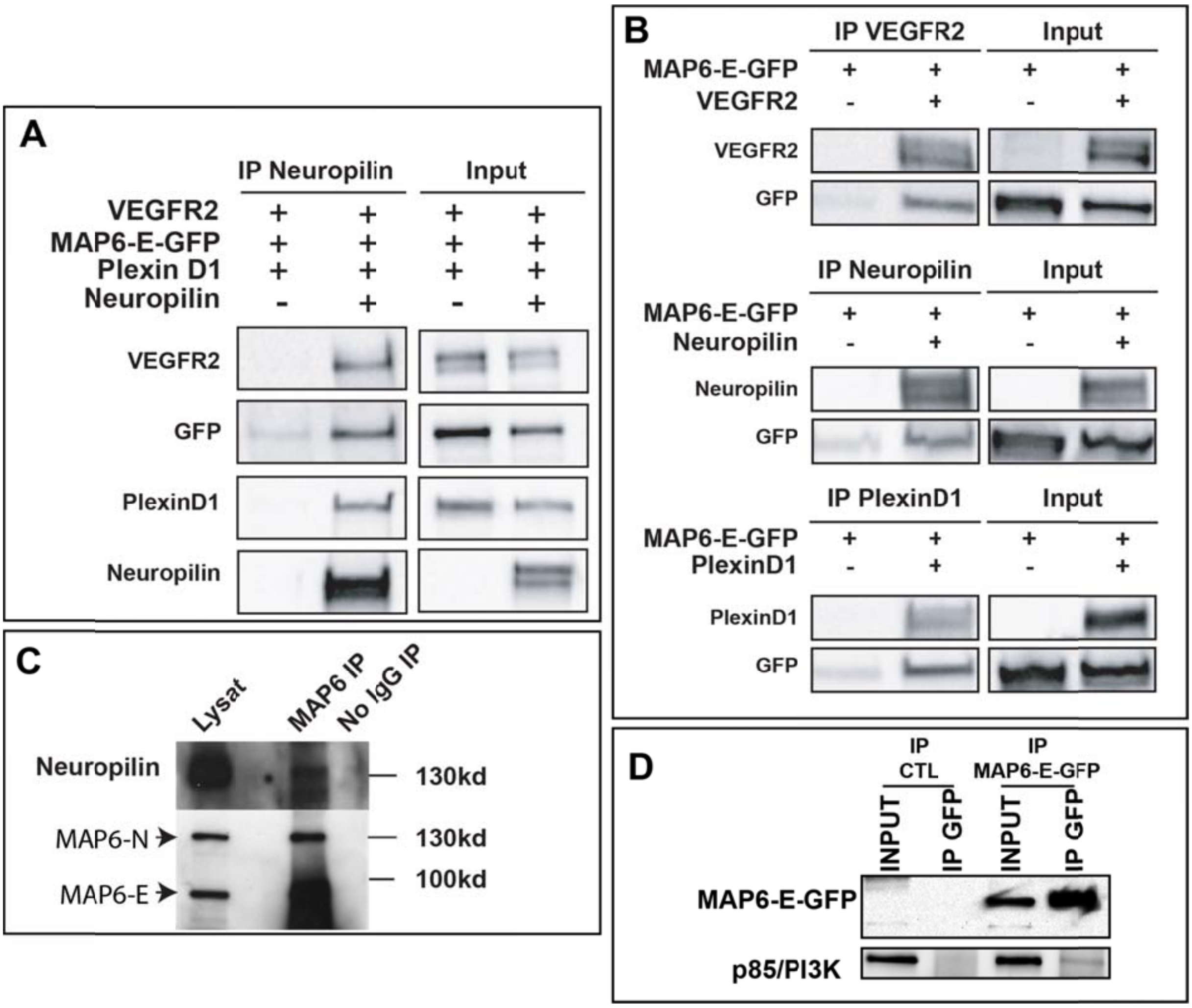

(A) HEK 293T17 cells were transfected with MAP6–E-GFP, VEGFR2, Plexin D1 and Neuropilin1 cDNAs. Immunoprecipitation was performed using polyclonal anti-Nrp1 antibody. PlxD1, VEGFR2 and MAP6 were found co-immunoprecipitated with Nrp1. In control experiments, Nrp1 cDNA was omitted.

(B) Immunoprecipitation of MAP6-E-GFP and Sema3E receptor proteins. HEK 293T17 cells were transfected with MAP6–E-GFP and either VEGFR2, PlexinD1 or Neuropilin1 cDNAs. MAP6 co-immunoprecipitated individually with PlxD1, VEGFR2 and Nrp1.

(C) Immunoprecipitation of endogenous MAP6-E and –N from subicular neurons using anti-MAP6 23N antibody. Endogenous Nrp1 was found immunoprecipitated with MAP6 proteins.

(D) HEK 293T17 cells were transfected with MAP6–E-GFP and immunoprecipitated using anti GFP antibody. Endogenous p85/PI3K was found in the immunoprecipitated.

Supplementary Figure 8: Subcellular localization of MAP6-E-GFP constructs in NIH-3T3 cells

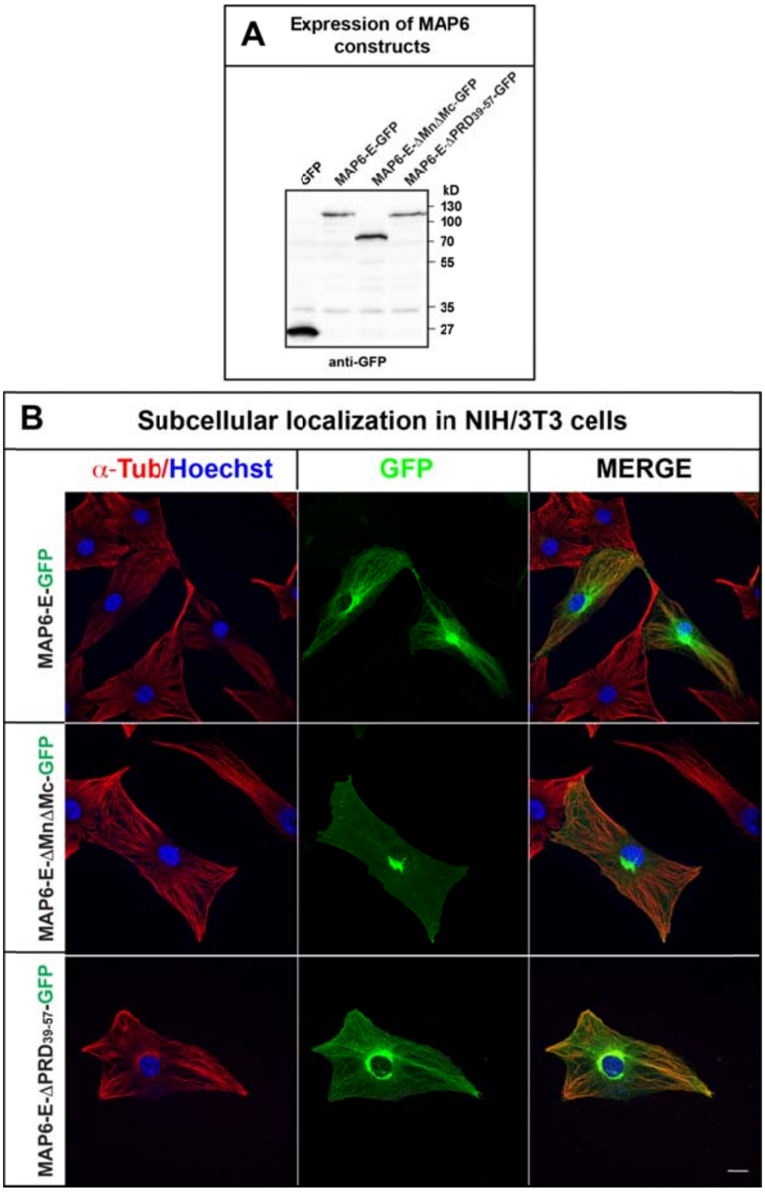

(A) Western blot analysis showing the expression of MAP6 constructs after transfection in subicular neurons.

(B) Subcellular localization of MAP6-E (upper panel), MAP6-E-ΔMnΔMc (middle panel) and MAP6-E-Δ $PRD_{39\text{-}42}$ fused with GFP in transfected NIH-3T3 cells. Cells were transfected using Lipofectamine 2000, fixed 18 h after transfection for 25 min with PFS (4% paraformaldehyde, 4% sucrose), permeabilized 3 min with PBS 0.2% Triton X-100, incubated with an anti tubulin antibody for 45 min in PBS-Tween 0.2% and with secondary antibodies for 40 min. Cells were analyzed with a confocal microscope LSM 710 (Zeiss). The deletion of Mc and Mn microtubule binding domains of MAP6-E suppress it association with the microtubular network to favor Golgi and membrane localization.
